# Global trends in insufficient physical activity among adolescents: a pooled analysis of 298 population-based surveys with 1·6 million participants

**DOI:** 10.1016/S2352-4642(19)30323-2

**Published:** 2020-01

**Authors:** Regina Guthold, Gretchen A Stevens, Leanne M Riley, Fiona C Bull

**Affiliations:** a Maternal, Newborn, Child and Adolescent Health and Ageing Department, WHO, Geneva, Switzerland; b Department of Non-communicable Diseases, WHO, Geneva, Switzerland; c Health Promotion Department, WHO, Geneva, Switzerland; d Independent Researcher, Los Angeles, CA, USA; e School of Public Health, Imperial College London, UK; f Department of Sport and Exercise Science, University of Western Australia, Perth, WA, Australia

## Abstract

**Background:**

Physical activity has many health benefits for young people. In 2018, WHO launched More Active People for a Healthier World, a new global action on physical activity, including new targets of a 15% relative reduction of global prevalence of insufficient physical activity by 2030 among adolescents and adults. We describe current prevalence and trends of insufficient physical activity among school-going adolescents aged 11–17 years by country, region, and globally.

**Methods:**

We did a pooled analysis of cross-sectional survey data that were collected through random sampling with a sample size of at least 100 individuals, were representative of a national or defined subnational population, and reported prevalence of of insufficient physical activity by sex in adolescents. Prevalence had to be reported for at least three of the years of age within the 10–19-year age range. We estimated the prevalence of insufficient physical activity in school-going adolescents aged 11–17 years (combined and by sex) for individual countries, for four World Bank income groups, nine regions, and globally for the years 2001–16. To derive a standard definition of insufficient physical activity and to adjust for urban-only survey coverage, we used regression models. We estimated time trends using multilevel mixed-effects modelling.

**Findings:**

We used data from 298 school-based surveys from 146 countries, territories, and areas including 1·6 million students aged 11–17 years. Globally, in 2016, 81·0% (95% uncertainty interval 77·8–87·7) of students aged 11–17 years were insufficiently physically active (77·6% [76·1–80·4] of boys and 84·7% [83·0–88·2] of girls). Although prevalence of insufficient physical activity significantly decreased between 2001 and 2016 for boys (from 80·1% [78·3–81·6] in 2001), there was no significant change for girls (from 85·1% [83·1–88·0] in 2001). There was no clear pattern according to country income group: insufficient activity prevalence in 2016 was 84·9% (82·6–88·2) in low-income countries, 79·3% (77·2–87·5) in lower–middle-income countries, 83·9% (79·5–89·2) in upper–middle-income countries, and 79·4% (74·0–86·2) in high-income countries. The region with the highest prevalence of insufficient activity in 2016 was high-income Asia Pacific for both boys (89·0%, 62·8–92·2) and girls (95·6%, 73·7–97·9). The regions with the lowest prevalence were high-income western countries for boys (72·1%, 71·1–73·6), and south Asia for girls (77·5%, 72·8–89·3). In 2016, 27 countries had a prevalence of insufficient activity of 90% or more for girls, whereas this was the case for two countries for boys.

**Interpretation:**

The majority of adolescents do not meet current physical activity guidelines. Urgent scaling up of implementation of known effective policies and programmes is needed to increase activity in adolescents. Investment and leadership at all levels to intervene on the multiple causes and inequities that might perpetuate the low participation in physical activity and sex differences, as well as engagement of youth themselves, will be vital to strengthen the opportunities for physical activity in all communities. Such action will improve the health of this and future young generations and support achieving the 2030 Sustainable Development Goals.

**Funding:**

WHO.

## Introduction

The health benefits of a physically active lifestyle during adolescence are well documented. They include improved cardiorespiratory and muscular fitness, bone and cardiometabolic health, and positive effects on weight status.^1^ Current evidence suggests that many of those health benefits carry forward into adulthood.^1^, ^2^ Furthermore, there is a growing body of evidence suggesting a positive impact of physical activity on cognitive development and prosocial behaviour.^3^

**Research in context**

**Evidence before this study**

Previous research showed that in 2010, the global prevalence of insufficient physical activity among school-going adolescents was 78·4% for boys and 84·4% for girls aged 11–17 years. With national device-based data (such as accelerometer or pedometer data) largely being absent or, when available, non-comparable across studies, these 2010 estimates were based on self-reported data from 120 countries. National data, when available, mostly confirmed high levels of insufficient activity in adolescents and additionally, a few data sets, mostly from high-income countries, typically showed no progress in decreasing levels of insufficient activity over time. However, information on global or regional time trends has been lacking, since too few countries had collected data using the same instrument in different years. Building on data included in these 2010 estimates, we obtained and added more recent data from existing WHO survey databases; through communication with WHO regional focal points, researchers and experts; verification of results of the 2017 NCD Country Capacity Survey on adolescent survey implementation; and a non-systematic literature search in PubMed (from database inception to Dec 31, 2016) focused on countries with no data, including the search terms “physical activity”, “adolescent”, and “school survey” (full list available in the appendix 3 p 11). We also had a country consultation on the preliminary set of estimates, closing in September, 2017, during which countries could submit additional data.

**Added value of this study**

This study presents updated country, regional, and global estimates of adolescent prevalence of insufficient physical activity, and reports, for the first time, global, regional, and national trends from 2001 to 2016, based on 298 surveys from 146 countries.

**Implications of all the available evidence**

Consistent with available evidence, our data show that levels of insufficient activity among adolescents continue to be extremely high, compromising their current and future health, and that small progress towards achieving the global target of a reduction of adolescent insufficient activity by 15% by 2030 has been made in boys, but not in girls. There is an urgent need for national and global action aimed at decreasing levels of insufficient activity, with a particular focus on adolescent girls, requiring strong government and stakeholder leadership to support the scaling of responses across multiple sectors. Our data will help guide national planning of policy actions, with a particular focus on reducing inequalities between adolescent girls and boys.

Current evidence also highlights the need for more harmonised, device-based physical activity data; more detailed physical activity data for different domains such as walking and cycling, sport, physical education, or unstructured activity during leisure time; and more disaggregated data for population subgroups. With these data, evidence-based interventions presented in the Global Physical Activity Action Plan 2018–30 can be better targeted and will improve population health and deliver co-benefits towards achieving many of the 2030 Sustainable Development Goals.

To achieve these benefits, international recommendations from WHO^2^ and 2018 US guidelines^1^ call for adolescents to do 60 min or more of daily physical activity of moderate-to-vigorous intensity.

In 2008, the global prevalence of school-going adolescents aged 13–15 years not meeting these recommendations was 80·3%.^4^ This value was the first global estimate based on an international compilation of adolescent physical activity data, using information from school-based surveys from 105 countries. Updated estimates for the year 2010 for school-going adolescents aged 11–17 years, including information from an additional 15 countries, showed a similar global prevalence of 78·4% for boys and 84·4% for girls.^5^ However, information on time trends was not included in these estimates, and has been previously identified as an area needing further research.^5^

Recognising the importance and urgency of reducing global levels of insufficient physical activity, WHO member states endorsed a global action plan on physical activity (GAPPA)^6^ and, at the World Health Assembly in 2018, agreed to a new target of a 15% relative reduction in insufficient physical activity among adolescents by 2030.

To support countries with this agenda, this paper presents adolescent prevalence of insufficient physical activity and estimates, for the first time, global, regional, and national trends from 2001 to 2016.^4^, ^5^

## Methods

### Study design and participants

We did a pooled analysis of pre-existing survey data (appendix 3 pp 11, 12). We included data fulfilling the following criteria: cross-sectional survey data from the general or school-going adolescent population; data collected through random sampling with a sample size of at least 100 individuals and representative of a national or defined subnational population; and prevalence of insufficient physical activity in adolescents reported by sex for at least three of the years of age within the 10–19-year age range, defined as not reaching the current WHO recommendation^2^ (doing less than 60 min of daily physical activity of moderate-to-vigorous intensity) or as being active for less than 60 min on 5 days per week. Survey questions were typically requesting respondents to report the number of days in a week they had been active, across all domains, for at least 60 minutes, and were largely based on a previously developed instrument^7^ that has been tested in diverse settings.^8^, ^9^ Physical activity data collected using wearable devices, such as accelerometers or pedometers, were not included because of limited comparability with self-reported data and absence of consensus of methods for data harmonisation across different devices.^10^, ^11^

When available, we used individual-record data to calculate the prevalence of insufficient physical activity, taking the sampling design into account. When raw data were not available, we used aggregated data as reported.

All data that met the inclusion criteria and that were provided before Sept 30, 2017, were included. Since all included surveys were done in the school setting, our estimates represent the school-going adolescent population.

### Procedures

We obtained data from existing WHO surveys and other multi-country surveys (for example, the Global School-based Student Health Survey [GSHS], and the Health Behaviour among School-aged Children [HBSC]). We also did a non-systematic literature search, focused on countries with no data. To identify additional data sources, we analysed and verified results of the 2017 WHO non-communicable diseases Country Capacity Survey, in which each WHO member state answered a question on the inclusion of questions on adolescent physical activity in national risk-factor surveys. We also had personal communications with WHO regional focal points, personal networks, and directly with researchers, including inquiries about additional data from authors of published studies. Finally, we did a 6-week consultation with all WHO member states, during which countries commented on the first draft set of estimates and submitted any additional data. After the consultation, the estimates were updated during October, 2017, with new data that were submitted.

We used the data from the included surveys to estimate the prevalence of insufficient physical activity in school-going adolescents aged 11–17 years (combined and by sex) for individual countries, for four World Bank income groups, nine regions,^12^, ^13^ and globally for the years 2001–16 (appendix 3 p 1). The World Bank income groups are: low, lower–middle, upper–middle, and high income. The nine regions are: central Asia, Middle East, and North Africa; central and eastern Europe; east and southeast Asia; high-income Asia Pacific; high-income western countries; Latin America and the Caribbean; Oceania; south Asia; and sub-Saharan Africa. Insufficient physical activity was defined as not meeting the WHO recommendations on physical activity for health:^2^ adolescents should, for health purposes, do at least 60 min of daily physical activity of moderate-to-vigorous intensity.

### Statistical analysis

Because of differences in study design, survey data were sometimes not comparable. We applied two key adjustments to survey data using linear regression modelling techniques to improve comparability. The first was a definition conversion. For surveys in which data were only reported for the definition of doing less than 60 minutes of physical activity on less than five days per week, and not for the current recommendation of seven days per week,^2^ we converted data in these sources to the current recommendation—our target indicator—using surveys reporting both definitions. The second adjustment was for surveys that only included data from urban populations (five surveys). For these surveys, we estimated prevalence in rural areas using information from surveys reporting both urban and rural prevalence. A national estimate combining the two was computed by applying estimates of population by area of residence for the respective survey year from the UN Population Division.

Of the 146 countries included, 73 had done at least two comparable surveys from different years between 2001 and 2016, using the same questionnaire (appendix 3 p 10). Using these trend data, we estimated the prevalence of insufficient physical activity for each year between 2001 and 2016 for all 146 countries, with a multilevel mixed-effects linear regression model. To allow estimates to be informed by data from the same country, from other countries in the region, and other variables, this model included a random slope on year, a random intercept for each country, fixed effects for country urbanisation, and location within nine previously defined regions that have been used in similar analyses.^12^, ^13^

To produce global and regional estimates and estimates for World Bank income groups, we created population-weighted sex-specific estimates for each subgroup and estimation year. Consistent with previously used methods for developing global estimates,^12^, ^14^ we used the bootstrap methodology and drew 1000 samples, each containing 80% of all survey data, to produce uncertainty intervals (UIs) for these estimates, representing the 2·5th and 97·5th percentile of the 1000 draws. To test for significance of trends and sex differences, we calculated the difference in prevalence between 2001 and 2016 and between sexes for each draw. We considered a change in prevalence over time or between sexes to be significant if at least 97·5% of draws showed a changing trend or a sex difference in the same direction.^12^, ^14^

Further methodological details are provided in appendix 3 pp 11–17.

### Role of the funding source

The funder of the study played no role in study design, data collection, data analysis, data interpretation, or writing of the report. All authors had full access to all the data in the study and the corresponding author had final responsibility for the decision to submit for publication.

## Results

We included 298 school-based surveys from 146 countries, territories, and areas (appendix 3 pp 2–9), representing 1·6 million students aged 11–17 years (81·3% of the global population of adolescents of this age). Data availability rose with country income, from 8 (26%) of 31 low-income countries with data available to 56 (75%) of 75 high-income countries with data available. Regional data availability ranged from 16 (30%) of 53 countries with data in Sub-Saharan Africa to 6 (100%) of 6 countries in south Asia (table 1).

**Table 1:** Distribution of data sources across income groups and regions

	**All countries, territories, and areas**	Income group*				**Region**								
		Low income	Lower–middle income	Upper–middle income	High income	Central Asia, Middle East, and north Africa	Central and eastern Europe	East and southeast Asia	High-income Asia Pacific	High-income western countries	Latin America and Caribbean	Oceania	South Asia	Sub-Saharan Africa
All countries	231	31	51	58	75	28	20	16	3	36	47	22	6	53
Countries, territories, and areas with data	146 (63%)	8 (26%)	37 (73%)	40 (69%)	56 (75%)	19 (68%)	16 (80%)	14 (88%)	2 (67%)	25 (69%)	32 (68%)	16 (73%)	6 (100%)	16 (30%)
Proportion of population covered by data	81%	36%	88%	89%	86%	71%	92%	98%	34%	100%	92%	23%	100%	30%
Countries, territories, and areas with trend data†	73 (32%)	2 (6%)	12 (24%)	17 (29%)	42 (56%)	11 (39%)	14 (70%)	5 (31%)	1 (33%)	25 (69%)	9 (19%)	2 (9%)	0	6 (11%)

Data are n, n (%), or %.

* 16 countries, territories, or areas are not classified into any income group by the World Bank.

† Percentage calculated over all countries, territories, and areas.

73 (50%) of 146 countries, territories, and areas with data (ie, 31·6% of all 231 countries globally) had trend data, defined as data from at least 2 different years between 2001 and 2016. Trend-data availability increased with country income and ranged across regions from no countries with trend data in south Asia to 14 (70%) of 20 countries in central and eastern Europe (table 1). Data availability was relatively evenly distributed across time, with 91 (31%) of 298 surveys from 2006 or earlier, 104 (35%) from 2007–2011, and 103 (35%) from 2012 or later (appendix 3 pp 2–9).

**Figure 1:**
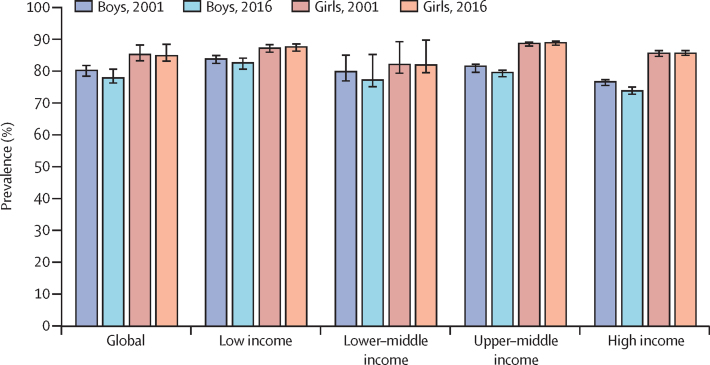
Prevalence of insufficient physical activity among school-going adolescents aged 11–17 years, globally and by World Bank income group, 2001 and 2016

More than four in five school-going adolescents aged 11–17 years were insufficiently physically active in 2016 (81·0% [95% UI 77·8–87·7]). Between 2001 and 2016, prevalence decreased by 2·5 percentage points (significant change) for boys (from 80·1% [78·3–81·6] to 77·6% [76·1–80·4]), whereas there was no significant change for girls (from 85·1% [83·1–88·0] to 84·7% [83·0–88·2]; figure 1), leading to a significant global difference of 7·1 percentage points in insufficient activity between sexes in 2016. If these trends continue, the global target of a 15% relative reduction in insufficient physical activity will not be met by 2030.

There was no clear pattern in prevalence according to country income group: insufficient physical activity was 84·9% (95% UI 82·6–88·2) in low-income countries, 79·3% (77·2–87·5) in lower–middle-income countries, 83·9% (79·5–89·2) in upper–middle-income countries, and 79·4% (74·0–86·2) in high-income countries in 2016. Although prevalence of insufficient activity showed small but significant decreases for boys of all income groups between 2001 and 2016 (except for lower–middle income), there were no decreases in any income group for girls, leading to widening differences in insufficient activity levels between sexes over time. In 2016, differences in insufficient activity between sexes ranged from 4·7 percentage points in lower–middle-income countries to 11·8 percentage points in high-income countries (figure 1).

High-income Asia Pacific was the region with the highest prevalence of insufficient physical activity in 2016 for both boys (89·0% [95% UI 62·8–92·2]) and girls (95·6% [73·7–97·9]). Sub-Saharan Africa was the region with the second highest prevalence of insufficient activity among boys (83·9% [82·3–85·0]), whereas for girls the region with the second highest prevalence was central Asia, Middle East, and north Africa (89·9% [88·6–90·9]). By contrast, high-income western countries (72·1% [71·1–73·6]) and south Asia (73·1 [69·3–86·9]) showed lowest prevalence in boys, and south Asia in girls (77·5% [72·8–89·3]; table 2).

**Table 2:** Estimated prevalence of insufficient physical activity among school-going adolescents aged 11–17 years by sex and overall, 2001 and 2016

	**Prevalence of insufficient physical activity (95% UI) in boys, 2001**	**Prevalence of insufficient physical activity (95% UI) in boys, 2016**	**Prevalence of insufficient physical activity (95% UI) in girls, 2001**	**Prevalence of insufficient physical activity (95% UI) in girls, 2016**	**Overall prevalence of insufficient physical activity (95% UI) in adolescents, 2001**	**Overall prevalence of insufficient physical activity (95% UI) in adolescents, 2016**
**Central and eastern Europe**						
Regional average	76·6% (75·6–77·6)	76·2% (74·5–77·3)	85·8% (84·8–86·7)	85·9% (84·5–86·8)	81·1% (76·7–86·5)	80·9% (76·3–86·6)
Albania	72·0% (68·3–75·4)	67·6% (63·4–71·5)	80·3% (76·9–83·3)	80·6% (77·3–83·5)	76·1% (72·5–79·3)	73·9% (70·1–77·3)
Bulgaria	71·3% (67·8–74·6)	67·0% (62·8–71·0)	80·3% (77·0–83·2)	80·1% (76·6–83·1)	75·7% (72·3–78·8)	73·3% (69·5–76·8)
Croatia	73·1% (69·8–76·2)	70·1% (66·1–73·7)	83·9% (81·1–86·4)	84·0% (81·1–86·5)	78·4% (75·3–81·2)	76·8% (73·4–79·9)
Czech Republic	74·2% (70·9–77·3)	73·1% (69·3–76·5)	82·2% (79·0–84·9)	82·0% (78·8–84·8)	78·1% (74·9–81·0)	77·4% (74·0–80·5)
Estonia	79·1% (76·2–81·7)	80·6% (77·6–83·3)	87·3% (85·0–89·3)	87·7% (85·4–89·7)	83·0% (80·4–85·4)	84·1% (81·4–86·4)
Hungary	75·0% (71·9–78·0)	73·5% (69·8–76·9)	85·6% (83·1–87·9)	85·8% (83·1–88·0)	80·2% (77·4–82·8)	79·5% (76·3–82·3)
Latvia	76·2% (73·1–79·0)	76·2% (72·7–79·3)	84·3% (81·5–86·7)	84·2% (81·4–86·6)	80·1% (77·2–82·8)	80·1% (76·9–82·9)
Lithuania	75·9% (72·8–78·8)	76·0% (72·5–79·2)	84·1% (81·4–86·6)	84·5% (81·8–86·9)	80·0% (77·0–82·6)	80·1% (77·0–82·9)
Moldova	74·8% (71·4–77·9)	73·3% (69·3–76·9)	78·8% (75·2–81·9)	78·2% (74·4–81·5)	76·7% (73·3–79·9)	75·7% (71·8–79·2)
North Macedonia	75·5% (72·4–78·4)	73·5% (69·8–77·0)	83·9% (81·1–86·3)	83·5% (80·6–86·1)	79·6% (76·6–82·2)	78·4% (75·1–81·4)
Poland	75·2% (72·1–78·1)	73·7% (70·0–77·1)	84·3% (81·6–86·7)	84·2% (81·4–86·7)	79·7% (76·7–82·3)	78·8% (75·6–81·8)
Romania	74·6% (71·3–77·6)	72·7% (68·9–76·2)	86·5% (84·1–88·6)	86·7% (84·3–88·9)	80·4% (77·5–83·0)	79·5% (76·4–82·4)
Russia	79·1% (76·2–81·8)	80·9% (77·9–83·6)	88·0% (85·7–89·9)	88·3% (86·1–90·3)	83·5% (80·9–85·8)	84·5% (81·9–86·8)
Slovakia	69·8% (66·3–73·2)	65·5% (61·2–69·6)	77·8% (74·3–81·0)	77·8% (74·1–81·1)	73·7% (70·2–77·0)	71·5% (67·5–75·2)
Slovenia	75·4% (72·1–78·3)	74·8% (71·0–78·2)	85·1% (82·4–87·4)	85·6% (82·8–87·9)	80·1% (77·1–82·8)	80·0% (76·8–82·9)
Ukraine	73·4% (70·1–76·5)	70·6% (66·7–74·3)	83·5% (80·6–86·0)	83·0% (80·0–85·6)	78·3% (75·2–81·1)	76·7% (73·2–79·8)
**Central Asia, Middle East, and north Africa**						
Regional average	80·6% (79·6–81·8)	80·2% (79·1–81·2)	89·6% (88·0–90·8)	89·9% (88·6–90·9)	85·0% (81·1–90·5)	85·0% (80·6–90·8)
Algeria	78·6% (75·3–81·7)	76·4% (73·0–79·6)	90·8% (88·9–92·3)	91·3% (89·7–92·7)	84·6% (82·0–86·9)	83·8% (81·2–86·1)
Armenia	76·8% (73·3–79·9)	73·0% (69·2–76·5)	83·5% (80·5–86·1)	82·8% (79·8–85·4)	80·1% (76·9–83·0)	77·7% (74·3–80·8)
Bahrain	77·7% (74·1–81·0)	75·0% (71·3–78·4)	87·6% (85·1–89·7)	87·4% (85·0–89·5)	82·4% (79·3–85·1)	81·0% (77·9–83·7)
Egypt	81·9% (78·7–84·8)	82·1% (78·9–84·9)	92·8% (91·2–94·1)	93·1% (91·6–94·4)	87·2% (84·8–89·3)	87·5% (85·1–89·5)
Iraq	80·4% (77·2–83·2)	79·9% (76·7–82·7)	90·2% (88·2–91·8)	90·5% (88·7–92·0)	85·1% (82·6–87·4)	85·0% (82·5–87·2)
Jordan	81·2% (78·1–83·9)	81·3% (78·3–84·0)	88·2% (85·9–90·2)	88·4% (86·2–90·3)	84·6% (81·9–87·0)	84·8% (82·2–87·1)
Kuwait	79·9% (76·4–83·0)	79·4% (76·0–82·5)	89·9% (87·7–91·8)	90·0% (87·9–91·7)	84·6% (81·7–87·2)	84·3% (81·5–86·8)
Lebanon	78·0% (74·5–81·2)	76·0% (72·4–79·2)	87·8% (85·3–89·8)	87·9% (85·6–89·9)	82·9% (79·9–85·5)	82·1% (79·1–84·7)
Libya	79·3% (76·0–82·3)	78·0% (74·6–81·0)	88·5% (86·2–90·4)	88·6% (86·5–90·4)	83·8% (81·0–86·2)	83·2% (80·4–85·6)
Mongolia	77·6% (74·1–80·7)	74·2% (70·5–77·5)	83·6% (80·6–86·2)	83·4% (80·6–85·9)	80·6% (77·4–83·4)	78·7% (75·5–81·7)
Morocco	83·1% (80·2–85·6)	84·6% (82·0–86·8)	89·5% (87·4–91·3)	90·1% (88·2–91·7)	86·2% (83·7–88·4)	87·3% (85·0–89·2)
Occupied Palestinian territory, including east Jerusalem	80·2% (77·0–83·0)	79·5% (76·4–82·4)	88·4% (86·2–90·3)	88·6% (86·5–90·4)	84·2% (81·5–86·6)	84·0% (81·3–86·3)
Oman	78·8% (75·6–81·8)	78·3% (75·0–81·2)	89·3% (87·2–91·1)	89·8% (87·9–91·5)	84·0% (81·3–86·4)	83·8% (81·2–86·1)
Qatar	84·0% (81·0–86·6)	86·3% (83·7–88·5)	90·6% (88·5–92·3)	90·9% (89·0–92·5)	87·0% (84·5–89·2)	88·2% (85·9–90·2)
Syria	82·9% (79·9–85·5)	84·1% (81·4–86·5)	90·7% (88·7–92·3)	91·1% (89·4–92·6)	86·7% (84·2–88·8)	87·5% (85·3–89·5)
Tunisia	77·7% (74·2–80·8)	74·9% (71·2–78·2)	88·3% (86·0–90·2)	88·4% (86·3–90·3)	82·9% (80·0–85·4)	81·5% (78·6–84·1)
Turkey	78·7% (75·3–81·6)	76·6% (73·2–79·8)	85·9% (83·3–88·2)	86·1% (83·6–88·3)	82·3% (79·3–84·9)	81·3% (78·3–83·9)
United Arab Emirates	79·1% (75·8–82·1)	77·9% (74·6–81·0)	86·7% (84·1–88·9)	86·9% (84·5–89·0)	82·6% (79·6–85·2)	81·9% (79·0–84·6)
Yemen	82·5% (78·9–85·5)	83·3% (80·1–86·1)	89·0% (86·4–91·2)	89·6% (87·2–91·6)	85·7% (82·6–88·3)	86·4% (83·6–88·8)
**East and southeast Asia**						
Regional average	83·1% (82·0–85·6)	82·0% (81·1–85·1)	88·8% (88·0–90·2)	89·3% (88·5–90·2)	85·9% (83·5–89·9)	85·5% (82·5–90·1)
Brunei	82·7% (79·0–85·9)	81·1% (77·1–84·4)	93·1% (91·2–94·6)	93·5% (91·8–94·9)	87·8% (85·0–90·1)	87·1% (84·3–89·5)
Cambodia	87·8% (85·1–90·1)	89·8% (87·5–91·7)	92·9% (91·1–94·4)	93·4% (91·8–94·7)	90·3% (88·1–92·2)	91·6% (89·6–93·2)
China	82·3% (78·8–85·3)	80·1% (76·3–83·4)	88·6% (86·0–90·8)	89·1% (86·7–91·1)	85·4% (82·3–88·0)	84·3% (81·1–87·0)
Taiwan	81·7% (77·8–85·0)	79·1% (74·9–82·8)	89·7% (87·1–91·9)	89·8% (87·3–91·9)	85·7 (82·5–88·4)	84·4 (81·1–87·3)
Indonesia	85·1% (82·1–87·7)	85·4% (82·5–87·9)	87·2% (84·4–89·6)	87·4% (84·7–89·7)	86·1% (83·2–88·6)	86·4% (83·6–88·8)
Laos	81·5% (77·8–84·7)	78·0% (74·1–81·5)	90·5% (88·2–92·4)	91·0% (89·0–92·7)	86·0% (83·0–88·5)	84·4% (81·5–87·0)
Malaysia	82·5% (78·9–85·6)	80·6% (76·6–84·0)	91·1% (88·8–92·9)	91·4% (89·3–93·2)	86·7% (83·7–89·2)	86·2% (83·2–88·7)
Maldives	81·3% (77·6–84·5)	77·9% (73·9–81·4)	86·0% (82·9–88·7)	86·1% (83·2–88·6)	83·6% (80·2–86·5)	81·9% (78·4–84·9)
Myanmar	84·3% (81·1–87·0)	84·1% (81·0–86·8)	89·2% (86·7–91·3)	89·6% (87·3–91·5)	86·8% (83·9–89·2)	86·8% (84·1–89·1)
Philippines	90·2% (88·1–92·0)	92·8% (91·2–94·1)	93·9% (92·4–95·2)	94·1% (92·7–95·2)	92·0% (90·2–93·5)	93·4% (91·9–94·6)
Sri Lanka	83·2% (79·7–86·2)	81·6% (77·9–84·8)	88·8% (86·0–91·0)	88·7% (86·0–90·9)	85·9% (82·8–88·6)	85·2% (82·0–87·9)
Thailand	77·8% (73·8–81·4)	70·2% (65·6–74·4)	85·3% (82·1–88·1)	85·0% (81·9–87·6)	81·5% (77·9–84·7)	77·5% (73·6–80·9)
Timor-Leste	85·3% (82·2–87·9)	85·5% (82·6–88·0)	92·8% (91·0–94·3)	93·4% (91·8–94·6)	89·0% (86·5–91·0)	89·4% (87·1–91·3)
Vietnam	83·5% (80·2–86·4)	82·3% (78·9–85·2)	90·3% (88·0–92·2)	90·6% (88·5–92·4)	86·9% (84·0–89·3)	86·3% (83·5–88·7)
**High-income Asia Pacific**						
Regional average	88·2% (64·2–92·7)	89·0% (62·8–92·2)	95·8% (74·0–97·9)	95·6% (73·7–97·9)	91·8 (86·4–97·8)	92·2 (87·2–97·9)
Singapore	77·7% (66·4–86·1)	69·7% (56·7–80·1)	84·9% (75·9–90·9)	83·1% (73·6–89·7)	81·2% (71·0–88·4)	76·3% (65·0–84·8)
South Korea	89·1% (82·2–93·5)	91·4% (85·8–94·9)	96·8% (94·4–98·2)	97·2% (95·2–98·4)	92·7% (88·0–95·7)	94·2% (90·3–96·6)
**High-income western countries**						
Regional average	75·4% (74·3–76·1)	72·1% (71·1–73·6)	84·5% (83·8–85·5)	84·6% (83·8–85·5)	79·8% (75·4–85·3)	78·2% (72·6–85·4)
Australia	83·5% (81·6–85·2)	86·8% (84·8–88·5)	90·8% (89·4–92·1)	91·4% (90·0–92·6)	87·0% (85·4–88·5)	89·0% (87·3–90·5)
Austria	74·7% (72·1–77·2)	71·2% (67·7–74·5)	84·3% (82·1–86·4)	84·5% (82·1–86·7)	79·4% (76·9–81·7)	77·8% (74·8–80·5)
Belgium	79·3% (76·9–81·5)	79·2% (76·4–81·8)	87·9% (86·0–89·6)	88·0% (86·0–89·7)	83·5% (81·4–85·5)	83·5% (81·1–85·7)
Canada	74·1% (71·5–76·6)	70·5% (67·1–73·7)	82·5% (80·1–84·6)	82·4% (79·8–84·7)	78·2% (75·7–80·5)	76·3% (73·3–79·0)
Denmark	80·4% (78·2–82·3)	82·1% (79·7–84·3)	86·3% (84·3–88·1)	87·0% (85·0–88·8)	83·2% (81·2–85·1)	84·5% (82·3–86·5)
Greenland	73·1% (70·4–75·6)	68·4% (64·8–71·7)	79·9% (77·3–82·3)	79·5% (76·6–82·1)	76·5 (73·8–79·0)	73·9 (70·7–76·9)
Finland	74·0% (71·4–76·4)	69·0% (65·6–72·3)	82·7% (80·4–84·9)	82·1% (79·6–84·5)	78·2% (75·8–80·5)	75·4% (72·4–78·2)
France	81·1% (79·0–83·0)	82·4% (80·0–84·6)	91·4% (90·1–92·6)	91·8% (90·4–93·0)	86·2% (84·5–87·7)	87·0% (85·1–88·7)
Germany	79·5% (77·3–81·6)	79·7% (77·0–82·1)	87·7% (85·9–89·3)	87·9% (86·0–89·6)	83·5% (81·5–85·3)	83·7% (81·4–85·8)
Greece	79·2% (77·0–81·3)	80·0% (77·4–82·5)	88·3% (86·6–89·8)	89·1% (87·4–90·6)	83·6% (81·6–85·4)	84·5% (82·3–86·4)
Iceland	77·0% (74·4–79·3)	75·4% (72·3–78·3)	85·3% (83·1–87·2)	85·2% (83·0–87·3)	81·0% (78·7–83·2)	80·3% (77·6–82·7)
Ireland	70·5% (67·4–73·4)	63·5% (59·5–67·3)	80·6% (77·8–83·1)	80·5% (77·6–83·2)	75·4% (72·5–78·1)	71·8% (68·3–75·0)
Israel	79·2% (76·9–81·4)	80·1% (77·4–82·5)	89·1% (87·4–90·6)	89·5% (87·8–91·0)	84·1% (82·0–85·9)	84·7% (82·5–86·7)
Italy	82·9% (80·9–84·7)	85·9% (83·9–87·8)	90·6% (89·2–91·9)	91·5% (90·0–92·7)	86·7% (84·9–88·2)	88·6% (86·8–90·2)
Luxembourg	76·3% (73·9–78·6)	73·4% (70·2–76·4)	85·5% (83·4–87·3)	85·4% (83·2–87·4)	80·8% (78·5–82·9)	79·2% (76·5–81·7)
Malta	77·3% (74·8–79·6)	76·7% (73·6–79·5)	85·7% (83·6–87·6)	85·8% (83·6–87·8)	81·4% (79·0–83·5)	81·4% (78·8–83·8)
Netherlands	77·5% (75·2–79·7)	76·6% (73·6–79·3)	83·4% (81·1–85·4)	83·9% (81·5–86·1)	80·4% (78·1–82·5)	80·2% (77·5–82·6)
New Zealand	82·5% (80·5–84·3)	84·9% (82·8–86·9)	92·1% (90·9–93·2)	92·7% (91·5–93·8)	87·2% (85·6–88·7)	88·7% (87·1–90·2)
Norway	79·3% (77·1–81·4)	78·6% (75·9–81·2)	88·3% (86·6–89·9)	88·6% (86·8–90·2)	83·7% (81·8–85·5)	83·5% (81·2–85·6)
Portugal	78·6% (75·9–81·0)	78·1% (75·1–80·8)	90·0% (88·3–91·5)	90·7% (89·1–92·1)	84·2% (82·0–86·1)	84·3% (82·0–86·4)
Spain	74·4% (71·9–76·8)	69·8% (66·4–73·0)	84·1% (81·9–86·0)	83·8% (81·4–86·0)	79·1% (76·7–81·3)	76·6% (73·7–79·3)
Sweden	80·6% (78·5–82·6)	82·2% (79·7–84·4)	86·8% (84·9–88·5)	87·3% (85·4–89·1)	83·6% (81·6–85·5)	84·7% (82·5–86·6)
Switzerland	80·9% (78·9–82·9)	82·5% (80·1–84·7)	88·5% (86·8–90·0)	89·1% (87·3–90·6)	84·6% (82·7–86·4)	85·7% (83·6–87·6)
UK	76·2% (73·8–78·5)	74·7% (71·6–77·5)	85·2% (83·2–87·1)	85·4% (83·2–87·4)	80·6% (78·4–82·7)	79·9% (77·3–82·3)
USA	70·9% (68·2–73·5)	64·0% (60·3–67·5)	80·7% (78·1–83·0)	80·5% (77·8–83·0)	75·7% (73·0–78·1)	72·0% (68·8–75·1)
**Latin America and Caribbean**						
Regional average	81·1% (80·0–82·2)	79·9% (78·8–81·4)	88·6% (87·6–89·6)	88·9% (87·9–89·8)	84·8% (81·0–89·4)	84·3% (79·9–89·6)
Anguilla	80·3% (76·8–83·3)	78·6% (75·2–81·7)	85·6% (82·6–88·1)	85·1% (82·3–87·6)	82·9% (79·7–85·7)	81·9% (78·7–84·6)
Antigua and Barbuda	78·1% (74·8–81·0)	73·9% (69·9–77·5)	85·0% (82·3–87·3)	84·6% (81·7–87·1)	81·7% (78·8–84·3)	79·2% (75·8–82·3)
Argentina	81·1% (78·1–83·8)	79·9% (76·9–82·6)	89·7% (87·7–91·4)	89·9% (88·0–91·5)	85·3% (82·8–87·5)	84·8% (82·3–87·0)
Bahamas	81·4% (78·6–83·9)	80·6% (77·9–83·1)	88·1% (86·0–89·9)	88·2% (86·3–89·9)	84·8% (82·3–86·9)	84·4% (82·0–86·5)
Barbados	79·6% (76·6–82·4)	76·9% (73·5–79·9)	86·9% (84·6–88·9)	86·9% (84·6–89·0)	83·2% (80·5–85·6)	81·8% (78·9–84·3)
Belize	79·3% (76·6–81·9)	76·4% (73·3–79·2)	84·5% (82·1–86·7)	84·2% (81·7–86·3)	81·9% (79·3–84·2)	80·3% (77·5–82·8)
Bolivia	82·5% (80·1–84·7)	82·4% (80·1–84·6)	88·3% (86·4–89·9)	88·6% (86·9–90·1)	85·4% (83·2–87·3)	85·5% (83·4–87·3)
Brazil	80·1% (77·2–82·7)	78·0% (75·0–80·8)	89·1% (87·2–90·8)	89·4% (87·6–91·0)	84·6% (82·1–86·7)	83·6% (81·1–85·8)
British Virgin Islands	79·9% (77·1–82·5)	77·5% (74·5–80·2)	86·7% (84·6–88·7)	86·9% (84·9–88·7)	83·3% (80·8–85·6)	82·2% (79·7–84·5)
Cayman Islands	81·3% (77·9–84·3)	80·6% (77·3–83·5)	88·8% (86·4–90·8)	88·9% (86·7–90·8)	85·1% (82·2–87·6)	84·8% (82·0–87·2)
Chile	83·9% (81·2–86·2)	84·2% (81·7–86·4)	90·9% (89·1–92·4)	91·2% (89·6–92·6)	87·3% (85·2–89·2)	87·6% (85·5–89·4)
Colombia	81·7% (79·1–84·1)	81·0% (78·4–83·4)	86·8% (84·7–88·7)	87·0% (84·9–88·8)	84·2% (81·9–86·3)	83·9% (81·6–86·0)
Costa Rica	79·2% (76·5–81·7)	76·1% (73·0–78·9)	87·6% (85·7–89·3)	88·2% (86·3–89·8)	83·3% (81·0–85·5)	82·0% (79·5–84·3)
Dominica	82·3% (79·9–84·6)	82·1% (79·7–84·3)	86·0% (83·8–87·9)	86·0% (84·0–87·9)	84·2% (81·8–86·2)	84·1% (81·8–86·1)
Ecuador	83·0% (80·6–85·1)	83·2% (80·9–85·3)	89·6% (87·9–91·1)	90·0% (88·4–91·3)	86·3% (84·2–88·1)	86·5% (84·6–88·2)
El Salvador	82·7% (80·4–84·9)	82·8% (80·5–84·9)	89·0% (87·3–90·6)	89·5% (87·9–90·9)	85·9% (83·8–87·7)	86·1% (84·1–87·9)
Grenada	82·3% (79·6–84·7)	81·8% (79·0–84·3)	86·8% (84·5–88·8)	86·9% (84·7–88·9)	84·5% (82·0–86·7)	84·3% (81·8–86·5)
Guatemala	83·6% (81·2–85·7)	84·5% (82·3–86·4)	88·9% (87·0–90·5)	89·4% (87·8–90·9)	86·2% (84·1–88·1)	86·9% (85·0–88·6)
Guyana	82·2% (79·4–84·8)	81·7% (78·7–84·3)	86·2% (83·7–88·4)	86·3% (83·8–88·5)	84·2% (81·5–86·6)	84·0% (81·3–86·4)
Honduras	81·4% (78·8–83·7)	80·2% (77·6–82·5)	87·2% (85·1–89·0)	87·6% (85·7–89·2)	84·3% (82·0–86·4)	83·8% (81·6–85·8)
Mexico	80·5% (77·8–83·0)	78·8% (76·0–81·4)	87·5% (85·4–89·3)	87·6% (85·7–89·4)	84·0% (81·6–86·1)	83·2% (80·8–85·3)
Montserrat	81·0% (77·1–84·4)	79·1% (75·1–82·7)	81·0% (76·8–84·6)	80·8% (76·7–84·3)	81·0% (76·9–84·5)	79·9% (75·9–83·5)
Netherlands Antilles	83·0% (80·5–85·2)	83·5% (81·2–85·6)	90·0% (88·2–91·5)	90·3% (88·8–91·7)	86·5% (84·4–88·3)	86·9% (85·0–88·6)
Paraguay	80·7% (78·1–83·0)	79·0% (76·3–81·4)	87·9% (86·0–89·6)	88·2% (86·4–89·7)	84·2% (82·0–86·2)	83·5% (81·3–85·5)
Peru	82·6% (80·1–84·9)	82·7% (80·3–84·9)	86·7% (84·5–88·6)	86·8% (84·8–88·7)	84·7% (82·3–86·7)	84·7% (82·5–86·8)
Saint Kitts and Nevis	80·5% (77·5–83·2)	78·5% (75·3–81·4)	86·1% (83·6–88·2)	86·1% (83·6–88·2)	83·3% (80·6–85·7)	82·3% (79·5–84·8)
Saint Lucia	82·8% (80·0–85·4)	82·8% (79·6–85·6)	86·1% (83·5–88·3)	85·9% (83·0–88·4)	84·5% (81·8–86·9)	84·3% (81·3–87·0)
Saint Vincent and the Grenadines	83·1% (80·6–85·3)	83·2% (80·9–85·3)	88·2% (86·2–89·9)	88·5% (86·7–90·1)	85·6% (83·4–87·6)	85·8% (83·8–87·7)
Suriname	80·3% (77·6–82·6)	78·4% (75·7–80·8)	84·9% (82·6–87·0)	84·6% (82·3–86·5)	82·5% (80·1–84·8)	81·4% (79·0–83·7)
Trinidad and Tobago	80·7% (77·1–83·9)	78·8% (74·7–82·4)	85·6% (82·5–88·3)	85·6% (82·3–88·4)	83·2% (79·8–86·1)	82·1% (78·5–85·3)
Uruguay	78·4% (75·0–81·5)	75·3% (71·7–78·6)	89·1% (86·9–90·9)	89·4% (87·4–91·1)	83·6% (80·8–86·1)	82·2% (79·3–84·7)
Venezuela	83·9% (81·2–86·3)	84·8% (82·3–86·9)	92·6% (91·1–93·8)	92·9% (91·6–94·0)	88·2% (86·0–90·0)	88·8% (86·8–90·4)
**Oceania**						
Regional average	82·9% (81·2–84·4)	81·9% (80·3–83·4)	86·1% (84·4–87·6)	86·2% (84·8–87·6)	84·5% (83·0–87·4)	84·0% (82·3–87·4)
American Samoa	83·7% (79·8–87·0)	83·7% (80·2–86·8)	87·7% (84·4–90·4)	87·8% (84·8–90·3)	85·7% (82·1–88·7)	85·8% (82·5–88·5)
Cook Islands	80·7% (76·8–84·1)	77·7% (73·6–81·4)	87·2% (84·3–89·7)	87·7% (85·0–90·0)	84·0% (80·6–86·9)	82·7% (79·3–85·7)
Fiji	82·4% (79·0–85·4)	80·7% (77·3–83·8)	86·0% (82·9–88·6)	86·0% (83·2–88·4)	84·2% (80·9–87·0)	83·3% (80·1–86·0)
French Polynesia	81·0% (77·3–84·2)	78·2% (74·4–81·6)	87·3% (84·5–89·7)	87·5% (85·0–89·7)	84·1% (80·8–86·9)	82·8% (79·6–85·6)
Guam	79·0% (74·2–83·1)	74·5% (69·4–79·0)	84·0% (79·9–87·5)	83·6% (79·7–87·0)	81·5% (77·0–85·3)	78·9% (74·4–82·9)
Kiribati	81·6% (77·9–84·7)	79·3% (75·6–82·6)	85·7% (82·7–88·4)	85·9% (83·0–88·3)	83·6% (80·2–86·5)	82·5% (79·2–85·4)
Nauru	83·6% (79·3–87·1)	83·6% (79·6–86·9)	89·7% (86·6–92·1)	90·0% (87·2–92·2)	86·6% (83·0–89·6)	86·8% (83·4–89·6)
Niue	85·4% (82·3–88·0)	86·1% (83·4–88·5)	87·9% (85·2–90·2)	88·5% (86·1–90·5)	86·6% (83·7–89·1)	87·3% (84·7–89·5)
Palau	79·8% (75·7–83·4)	75·9% (71·2–80·1)	82·1% (78·2–85·5)	82·1% (78·1–85·6)	81·0% (76·9–84·5)	79·0% (74·6–82·8)
Samoa	86·1% (83·0–88·8)	87·5% (84·6–89·8)	86·5% (83·4–89·2)	86·7% (83·6–89·2)	86·3% (83·2–88·9)	87·1% (84·1–89·5)
Solomon Islands	83·2% (79·5–86·3)	82·1% (78·5–85·2)	85·1% (81·6–88·1)	85·4% (82·2–88·1)	84·1% (80·5–87·2)	83·7% (80·3–86·6)
Tokelau	79·3% (74·5–83·4)	74·1% (68·7–78·8)	80·9% (76·1–84·8)	80·3% (75·6–84·3)	80·1% (75·3–84·1)	77·2% (72·1–81·6)
Tonga	85·7% (82·6–88·4)	86·7% (83·9–89·2)	84·8% (81·3–87·7)	84·8% (81·5–87·6)	85·3% (82·0–88·1)	85·8% (82·8–88·4)
Tuvalu	84·9% (81·7–87·5)	85·3% (82·4–87·8)	88·3% (85·6–90·5)	88·9% (86·6–90·9)	86·6% (83·7–89·0)	87·1% (84·5–89·4)
Vanuatu	85·4% (82·2–88·1)	86·2% (83·3–88·6)	88·5% (85·7–90·8)	89·0% (86·6–91·1)	86·9% (83·9–89·4)	87·5% (84·8–89·8)
Wallis and Futuna	84·5% (80·7–87·7)	84·6% (80·8–87·7)	86·4% (82·8–89·4)	86·8% (83·3–89·7)	85·5% (81·7–88·6)	85·7% (82·0–88·7)
**South Asia**						
Regional average	77·3% (72·0–87·3)	73·1% (69·3–86·9)	77·6% (72·6–89·0)	77·5% (72·8–89·3)	77·4% (72·8–83·5)	75·2% (71·9–83·8)
Afghanistan	85·9% (81·1–89·6)	88·5% (84·5–91·5)	86·9% (82·3–90·5)	87·8% (83·5–91·1)	86·4% (81·7–90·0)	88·1% (84·0–91·3)
Bangladesh	72·8% (65·4–79·1)	63·2% (55·0–70·6)	70·6% (62·6–77·5)	69·2% (61·3–76·1)	71·7% (64·0–78·3)	66·1% (58·1–73·3)
Bhutan	82·5% (76·9–86·9)	82·8% (77·4–87·1)	84·6% (79·3–88·7)	85·4% (80·5–89·3)	83·5% (78·1–87·8)	84·1% (78·9–88·2)
India	76·6% (69·7–82·3)	71·8% (64·4–78·2)	76·6% (69·5–82·5)	76·3% (69·4–82·1)	76·6% (69·6–82·4)	73·9% (66·8–80·0)
Nepal	81·9% (76·2–86·6)	81·8% (76·1–86·3)	84·6% (79·2–88·8)	85·3% (80·2–89·2)	83·3% (77·7–87·7)	83·5% (78·1–87·7)
Pakistan	84·0% (78·8–88·2)	85·4% (80·6–89·2)	87·9% (83·5–91·2)	88·6% (84·6–91·7)	85·9% (81·0–89·7)	87·0% (82·5–90·4)
**Sub-Saharan Africa**						
Regional average	84·9% (83·5–85·8)	83·9% (82·3–85·0)	88·4% (87·2–89·0)	88·5% (87·3–89·3)	86·6% (85·1–88·9)	86·2% (84·1–89·2)
Benin	78·6% (75·2–81·6)	71·3% (67·1–75·2)	81·5% (78·0–84·5)	80·7% (77·1–83·8)	80·0% (76·6–83·1)	75·9% (72·0–79·5)
Botswana	85·9% (83·3–88·2)	86·1% (83·4–88·4)	88·7% (86·3–90·7)	88·9% (86·5–90·9)	87·3% (84·8–89·4)	87·5% (84·9–89·6)
Djibouti	83·1% (79·6–86·1)	81·3% (77·5–84·5)	89·2% (86·5–91·4)	89·3% (86·7–91·4)	86·1% (83·1–88·7)	85·2% (82·0–87·9)
Ghana	86·2% (83·7–88·3)	86·6% (84·0–88·8)	88·1% (85·6–90·2)	88·4% (85·9–90·5)	87·1% (84·7–89·2)	87·5% (85·0–89·6)
Kenya	85·3% (82·6–87·7)	84·9% (82·0–87·4)	88·6% (86·1–90·7)	88·9% (86·4–90·9)	86·9% (84·3–89·2)	86·8% (84·2–89·1)
Mauritania	84·3% (81·5–86·7)	83·2% (80·1–85·9)	90·9% (88·9–92·5)	91·4% (89·4–93·0)	87·5% (85·2–89·6)	87·2% (84·7–89·4)
Mauritius	80·7% (77·6–83·6)	76·2% (72·4–79·6)	88·5% (86·1–90·5)	88·4% (86·0–90·5)	84·6% (81·8–87·0)	82·2% (79·1–84·9)
Mozambique	84·2% (81·5–86·6)	83·0% (79·9–85·6)	90·8% (88·8–92·4)	91·3% (89·4–92·9)	87·5% (85·2–89·6)	87·1% (84·6–89·2)
Namibia	86·4% (84·0–88·5)	86·5% (84·0–88·6)	88·1% (85·7–90·2)	88·4% (85·9–90·4)	87·3% (84·8–89·3)	87·4% (85·0–89·5)
Senegal	85·2% (82·5–87·5)	84·8% (82·0–87·2)	91·9% (90·1–93·3)	92·2% (90·5–93·7)	88·5% (86·3–90·4)	88·5% (86·2–90·4)
Seychelles	81·8% (78·7–84·5)	78·7% (75·1–81·9)	86·8% (84·1–89·1)	86·7% (84·0–89·1)	84·3% (81·4–86·8)	82·6% (79·5–85·4)
Sudan	88·0% (85·8–89·9)	89·6% (87·6–91·3)	90·6% (88·7–92·3)	91·0% (89·1–92·7)	89·3% (87·2–91·1)	90·3% (88·3–92·0)
Tanzania	81·9% (78·8–84·7)	78·2% (74·6–81·5)	86·0% (83·1–88·5)	86·0% (83·1–88·4)	84·0% (81·0–86·6)	82·1% (78·9–84·9)
Uganda	84·9% (81·9–87·4)	84·0% (80·8–86·8)	87·2% (84·3–89·6)	87·3% (84·5–89·7)	86·0% (83·1–88·5)	85·7% (82·7–88·2)
Zambia	88·1% (85·9–90·0)	89·4% (87·4–91·2)	88·8% (86·5–90·8)	89·1% (86·8–91·0)	88·5% (86·2–90·4)	89·3% (87·1–91·1)
Zimbabwe	85·1% (82·4–87·4)	84·6% (81·8–87·1)	88·6% (86·2–90·6)	88·6% (86·2–90·7)	86·8% (84·3–89·0)	86·6% (84·0–88·9)

**Figure 2:**
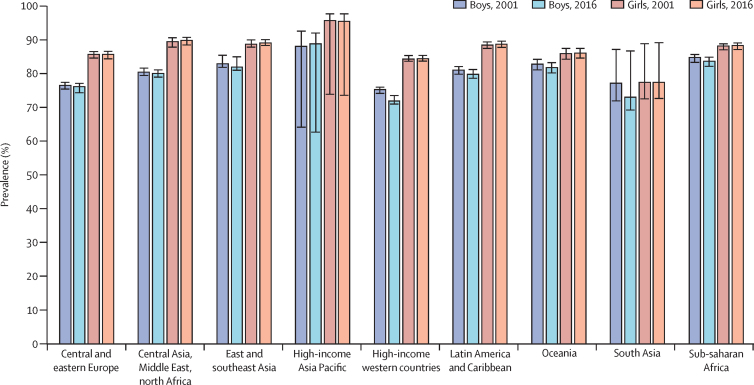
Prevalence of insufficient physical activity among school-going adolescents aged 11–17 years, by sex and region, 2001 and 2016

Across all regions, girls were less active than boys, with significant differences between sexes in seven of the nine regions. Insufficient physical activity did not change for girls or both sexes combined in any region; however, male prevalence showed small but significant decreases in five of the nine regions (high-income western countries, Latin America and the Caribbean, Oceania, south Asia, and sub-Saharan Africa; figure 2). As a result, with the exception of high-income Asia Pacific, differences between sexes were widening in all regions, resulting in a range of a 4·3 percentage-point difference in Oceania to a 12·5 percentage-point difference in high-income western countries in 2016 (figure 2).

**Figure 3:**
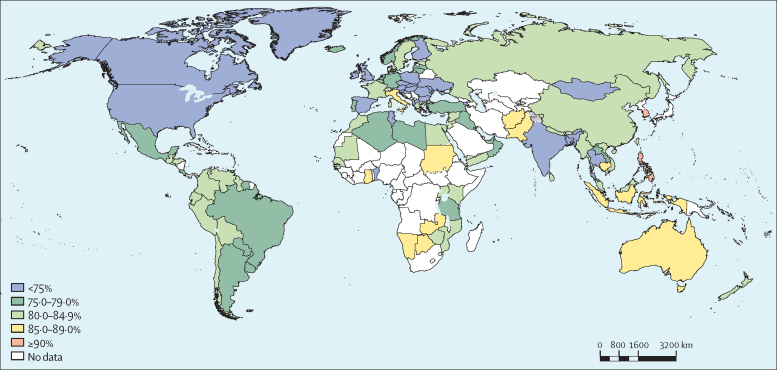
Prevalence of insufficient physical activity among school-going boys aged 11–17 years, 2016

**Figure 4:**
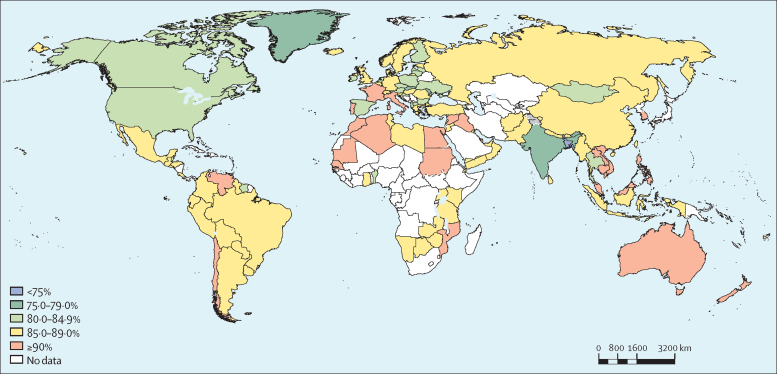
Prevalence of insufficient physical activity among school-going girls aged 11–17 years, 2016

In 2016, prevalence of insufficient physical activity was more than 80% in 71 (49%) of 146 countries analysed for boys versus 141 (97%) for girls, more than 85% in 20 (14%) countries for boys versus 112 (77%) countries for girls, and more than 90% in two (1%) countries for boys versus 27 (18%) countries for girls (table 2; Figure 3, Figure 4).

Philippines was the country with the highest prevalence of insufficient activity among boys (92·8 [91·2–94·1]), whereas South Korea showed highest levels among girls (97·2 [95·2–98·4]) and both sexes combined (94·2 [90·3–96·6]). Bangladesh was the country with the lowest prevalence of insufficient physical activity among boys (63·2 [55·0–70·6]), girls (69·2 [61·3–76·1]), and both sexes combined (66·1 [58·1–73·3]; table 2).

Girls were less active than boys in all but four (3%) countries (Tonga, Samoa, Afghanistan, and Zambia), in 2016. In 43 (29%) countries, the difference between sexes was greater than 10 percentage points and in the USA and Ireland it was greater than 15 percentage points (appendix 3 p 18).

Among boys, in 6 (4%) of the 146 countries in our analysis, prevalence of insufficient activity decreased by more than 5 percentage points since 2001 (Bangladesh, Singapore, Thailand, Benin, Ireland, and the USA), whereas in Italy and Australia it increased by more than 3 percentage points. Among girls, changes in insufficient activity prevalence over time were small, ranging from a 1·7 percentage-point decrease in insufficient activity in Singapore to a 0·9 percentage-point increase in Afghanistan. Differences in prevalence between sexes widened from 2001 to 2016 in most countries analysed (107 [73%] of 146; table 2).

## Discussion

Our analysis shows that globally, in 2016, more than 80% of school-going adolescents aged 11–17 years did not meet current recommendations for daily physical activity, compromising their current and future health.^2^ Although the prevalence of insufficient physical activity has slightly decreased in boys since 2001, there was no change over time in girls, and if these trends continue, the global target of a 15% relative reduction in insufficient physical activity—which would lead, if met, to a global prevalence of less than 70% by 2030—will not be achieved.

We found that globally, across income groups and regions and in nearly all countries analysed, girls were less active than boys and the prevalence of female insufficient activity, in particular, has not improved since 2001. Differences in prevalence of insufficient physical activity between boys and girls and widening gaps over time were particularly apparent in some high-income countries, such as Singapore, the USA, and Ireland, all showing an absolute difference in prevalence between boys and girls of more than 13 percentage points in 2016 (an increase in sex difference of more than 5 percentage points relative to 2001). These differences between sexes^15^ and widening gaps^16^ have been confirmed by several articles describing national data from these countries, as well as by objective, device-based data from other countries such as Canada with estimates similar to ours.^17^

A previous meta-analysis reported that changing physical activity behaviour, particularly in adolescent girls, is challenging; however, small effects of some interventions are reported, specifically those that were underpinned by theory and based on multicomponent strategies.^18^ Furthermore, there are examples of national campaigns that have effectively addressed specifically the gender gap (eg, This Girl Can campaign in the UK).^19^ Although the target population for this campaign is adult women, the visibility and creation of more active female role models can positively influence girls' decisions and participation. Social marketing campaigns combined with community-based interventions should be starting points to increase physical activity levels in girls, particularly in countries with wide differences between sexes. This approach has been identified as cost-effective,^20^ and is a core recommendation of the GAPPA.^6^

Unlike in adults,^12^ we did not find that prevalence of insufficient activity in school-going adolescents increased with country income. On the contrary, male adolescents from high-income countries showed the lowest prevalence of insufficient activity in our analysis, whereas those from low-income countries showed the highest prevalence. However, this was not the case for girls, for whom we did not find any pattern with regards to country income. Previous research on the effect of socioeconomic status on physical activity participation among adolescents indicates that adolescents with higher socio-economic status tended to be more active than those with lower socioeconomic status, perhaps because of more opportunities at school and in the community to be active for adolescent boys, whereas there might be fewer opportunities meeting the needs and interests of adolescent girls. However, findings on this social patterning across studies are not uniform,^21^ and more research is needed to improve the understanding of this relationship.

We found the highest levels of insufficient activity in boys in high-income Asia Pacific. Sub-Saharan Africa was the region with the second highest prevalence of insufficient activity among boys, with particularly high prevalence values in Sudan and Zambia. It must be noted that we only included school-going adolescents in our sample, which might represent not only a small proportion of this age group in these settings, but perhaps also a population with special characteristics. For example, these adolescents might be more likely to come from more advantaged households, that might be more focused on high achievement in other academic disciplines, rather than physical education, sports, and active recreation. The low priority given to physical activity within the context of some schools and parental attitudes is a frequently cited barrier as it reduces the support and opportunities available to adolescents.^15^, ^22^

Some of the lowest levels of insufficient activity in boys were found in high-income western countries and south Asia, driven by countries with large populations like the USA, Bangladesh, and India. The quite low prevalence of insufficient activity in boys in Bangladesh and India might be explained by the strong focus on national sports, such as cricket, which is frequently played unstructured in local communities. In the USA, these data might be explained by better physical education in schools, the pervasive media coverage of sports, and a strong presence of sports clubs providing many opportunities to play in structured, organised sport (such as ice hockey, American football, basketball, or baseball), often rather male-dominated activities.

For adolescent females, the high-income Asia Pacific region showed the highest levels of insufficient activity, largely driven by South Korea. The region with the lowest levels of insufficient activity in 2016 in girls was south Asia, which includes Bangladesh and India. These two countries reported the lowest prevalence in female insufficient activity in our study, potentially explained by societal factors, such as girls being required to support activity and domestic chores around the home.

Our results need to be interpreted in light of several limitations. First, we only included school-going adolescents in our analysis, and our results are therefore not representative of the entire adolescent population of each country. In some countries and regions, out-of-school adolescents represent a large population^23^ with potentially different behaviours as compared with school-going adolescents. Including out-of-school adolescents in our analysis would probably have influenced our findings but was not possible because of lack of data in this largely understudied population.^24^ We call for this gap in surveillance of physical activity to be urgently addressed to enable countries to respond and meet the call for reporting of disaggregated health statistics.^25^

Second, the surveys included in our analysis covered different years of the adolescent age range, which we aggregated into a single estimate for adolescents aged 11–17 years. Most importantly, the HBSC, done in many European and North American countries, includes adolescents aged 11 years, 13 years, and 15 years, whereas the GSHS focused on adolescents aged 13–15 years until 2012, and then expanded to adolescents aged 13–17 years. Our aggregation of all ages might have led to increased estimates of insufficient activity in countries where older age groups were included, since some national reports^15^, ^26^ show increases in insufficient activity during the adolescent years. However, in a study done in England, Farooq and colleagues^27^ found declines in physical activity occurred earlier (around the age of 7 years), and less so during adolescence. A recent review by Kemp and colleagues on longitudinal changes in domains of physical activity during childhood and adolescence^28^ showed no change in transport or in organised sport participation during adolescence, and concluded that most changes in physical activity behaviour are likely to happen before adolescence, while mentioning that changes in non-organised physical activity have been studied less. Notably, this review reported data by specific domains of physical activity, such as activity for transport, in school, in sport clubs, or unstructured active play, which was not possible in our global dataset in which we could only report on overall physical activity, because of non-comparable national survey data on other domains. This inconsistency in monitoring is also seen in many other areas of adolescent health measurement and calls for development of more standardised and harmonised tools for collection and reporting—an area that the Global Action for Measurement of Adolescent health is aiming to address.^24^

Third, we calculated our estimates from self-reported data, despite their known flaws,^10^, ^29^ including potential social desirability bias and cross-cultural, age, or sex differences in reporting. For instance, although examples of setting-specific activities were usually provided in the question text of surveys we included, active transportation and domestic chores might not always be included in reporting, potentially leading to biased estimates. Another flaw includes the sometimes-limited validity and reliability of survey instruments. Although questionnaires used in studies that were included in our analysis were tested for validity and reliability in different settings^7^, ^8^, ^9^ and have been recommended by experts,^30^ further testing with diverse populations and potential adaptation of questionnaires is needed.^8^ Using device-based, objective data for estimation of regional and global estimates of insufficient physical activity is currently not possible, since the availability of nationally representative, device-based measured physical activity data among adolescents is limited to a few, mainly high-income countries.^5^ Furthermore, despite repeated calls,^11^ there are no global standard protocols for analysing and reporting of device-based measurements, and different data cleaning methods and cut-points are applied in different studies, leading to non-comparability of results.^10^ In a recent study, Migueles and colleagues concluded that it is currently not possible to know the prevalence of meeting physical activity guidelines based on accelerometer data.^31^ Hence, until these issues have been resolved, self-report data from questionnaires that have been validated and recommended by experts,^7^, ^8^, ^9^, ^30^ such as those used in surveys included in our analysis, are the only option for this type of large global and regional analyses. The development of standardised methods and analysis protocols for device-based assessment of physical activity, such as continuing efforts by partners involved in the International Children's Accelerometry Database,^32^ remains an urgent priority for this field.

Finally, similar to all large global and regional analyses, data were not available for every country and year, and availability varied across countries and regions. Although available data covered more than 80% of the global population, the population of low-income countries was only covered by 36%. Trend-data availability was also skewed towards high-income countries, and our estimates for low income countries therefore need to be interpreted with caution.

In 2018, all countries committed to implementing the policy actions recommended in the GAPPA,^6^ and the data presented in this paper emphasise the urgent need for accelerating the speed and scale of national and subnational responses. WHO guidance^33^ recommends that all countries develop or update national policy and implementation plans on physical activity and, most importantly, allocate the necessary political priority and resources to enable implementation, or their commitment to increase physical activity will not be achieved. Without exception, all countries should prioritise policy and programmes that target children and adolescents, especially girls.

Effectively addressing the high prevalence of insufficient activity will require identifying, understanding, and intervening on the causes and inequities—social, economic, cultural, technological, and environmental—that can perpetuate the low levels of participation and differences between sexes. Policy actions should aim to address increasing physical activity in all its forms, including physical education that develops foundational physical literacy, sport, active play, and recreation, as well as safe independent mobility (walking and cycling). Comprehensive action will require engagement and coordinated responses across multiple sectors and stakeholders including, but not limited to, schools, families, sport and recreation providers, urban planners, and city and community leaders. To support countries, global and regional guidance on effective approaches exist,^6^, ^33^ and recent examples of new national policy with this agenda provide other countries with a practical template as well as useful advocacy material.^34^

In summary, our analysis, based on 1·6 million school-going adolescents, is the first to estimate levels of insufficient physical activity across 146 countries and to assess global, regional, and country time trends in insufficient physical activity. Our data show that the majority of adolescents do not meet physical activity guidelines, putting their current and future health at risk. Although there appear to have been small reductions in insufficient activity among boys, prevalence of insufficient physical activity in girls has remained unchanged since 2001, leading to widening sex differences. Urgent action is needed now, particularly through targeted interventions to promote and retain girls' participation in physical activity. Policy action aimed at increasing physical activity should be prioritised, and stronger government and stakeholder leadership is needed to support the scaling of responses across multiple sectors. Young people have the right to play and should be provided with the opportunities to realise their right to physical and mental health and wellbeing. That four in every five adolescents do not experience the enjoyment and social, physical, and mental health benefits of regular physical activity is not by chance, but a consequence of political choices and societal design. The contribution of policy actions that will increase physical activity will, at the same time, support achieving multiple Sustainable Development Goals. Policy makers and stakeholders should be encouraged to act now for the health of this and future young generations.

## Data sharing

The master dataset (including aggregated results by sex and age from each survey included in the analysis); the datasets including the results of the analyses by country, region, World Bank income group, and globally; and the data dictionaries for each dataset will be made available to others upon request via email to the corresponding author.

## Supplementary Materials

French translation of the abstract

Spanish translation of the abstract
