# Supplementary material for: Global trends in insufficient physical activity among adolescents: a pooled analysis of 298 population-based surveys with 1·6 million participants
Source: Lancet Child Adolesc Health. 2020 Jan;4(1):23–35. doi: 10.1016/S2352-4642(19)30323-2 (PMC6919336; doi:10.1016/S2352-4642(19)30323-2)
Supplement: French translation of the abstract [file mmc1.pdf]

THE LANCET
Child & Adolescent Health

Supplementary appendix 1

This translation in French was submitted by the authors and we reproduce it as supplied. It has not been peer reviewed. *The Lancet*'s editorial processes have only been applied to the original in English, which should serve as reference for this manuscript.

Supplement to: Guthold R, Stevens GA, Riley LM, Bull FC. Global trends in insufficient physical activity among adolescents: a pooled analysis of 298 population-based surveys with 1·6 million participants. *Lancet Child Adolesc Health* 2019; published online Nov 21. https://doi.org/10.1016/S2352-4642(19)30323-2.

Cette traduction en français a été proposée par les auteurs et nous l'avons reproduite telle quelle. Elle n'a pas été examinée par des pairs. Les processus éditoriaux de *Lancet* n'ont été appliqués qu'à l'original en anglais, ce qui devrait servir de référence à ce manuscrit.

Tendances mondiales de l'insuffisance d'activité physique chez les adolescents: analyse groupée de 298 enquêtes en population avec 1·6 million de participants.

Tendances mondiales de l'insuffisance d'activité physique chez les adolescents: analyse groupée de 298 enquêtes en population avec 1,6 million de participants

Synthèse

Contexte L'activité physique a de nombreux effets bénéfiques pour la santé des jeunes. En 2018, l'OMS a lancé un nouveau plan d'action mondial de promotion de l'activité physique, intitulé « Des personnes plus actives pour un monde plus sain », ciblant une réduction relative de 15 % de la prévalence mondiale de l'insuffisance d'activité physique d'ici à 2030 chez les adolescents et les adultes. Le présent article décrit la prévalence et les tendances actuelles de l'insuffisance d'activité physique chez les adolescents scolarisés âgés de 11 à 17 ans par pays, par région et à l'échelle mondiale.

Méthodes Nous avons effectué une analyse groupée de données issues d'enquêtes transversales, obtenues par échantillonnage aléatoire avec une taille d'échantillon d'au moins 100 personnes. Ces données devaient être représentatives d'une population nationale ou locale définie et fournir des informations sur la prévalence de l'insuffisance d'activité physique selon le sexe chez les adolescents. La prévalence devait être renseignée pour au moins 3 années dans la tranche d'âge comprise entre 10 et 19 ans. Nous avons estimé la prévalence de l'insuffisance d'activité physique chez les adolescents scolarisés âgés de 11 à 17 ans (selon le sexe et tous sexes confondus) par pays, pour quatre groupes de revenus définis par la Banque mondiale, pour neuf régions et à l'échelle mondiale dans la période 2001-2016. Pour obtenir une définition standard de l'insuffisance d'activité physique et pour tenir compte de la couverture des enquêtes menées uniquement en milieu urbain, nous avons utilisé des modèles de régression. Nous avons estimé les tendances dans le temps au moyen d'une modélisation à effets mixtes à plusieurs niveaux.

Résultats Nous avons utilisé les données issues de 298 enquêtes menées en milieu scolaire dans 146 pays, territoires et zones, portant sur 1,6 million d'élèves âgés de 11 à 17 ans. À l'échelle mondiale, en 2016, 81,0 % (intervalle de confiance à 95 % : 77,8–87,7) des élèves âgés de 11 à 17 ans avaient une activité physique insuffisante (77,6 % [76,1–80,4] des garçons et 84,7 % [83,0–88,2] des filles). Bien que cette prévalence ait enregistré une baisse significative entre 2001 et 2016 pour les garçons (80,1 % [78,3–81,6] en 2001), aucun changement significatif n'a été observé pour les filles (85,1 % [83,1–88,0] en 2001). Aucun lien évident n'a été constaté entre le groupe de revenu auquel appartenaient les pays et l'insuffisance de l'activité physique : en 2016, la prévalence de l'insuffisance d'activité physique était de 84,9 % (82,6–88,2) dans les pays à faible revenu, de 79,3 % (77,2–87,5) dans les pays à revenu intermédiaire de la tranche inférieure, de 83,9 % (79,5–89,2) dans les pays à revenu intermédiaire de la tranche supérieure et de 79,4 % (74,0–86,2) dans les pays à revenu élevé. La région affichant la plus forte prévalence d'insuffisance de l'activité physique en 2016 était la région Asie-Pacifique à revenu élevé, aussi bien pour les garçons (89,0 %, 62,8–92,2) que pour les filles (95,6 %, 73,7–97,9). La prévalence la

plus faible était observée dans les pays occidentaux à revenu élevé pour les garçons (72,1 %, 71,1–73,6) et en Asie du Sud pour les filles (77,5 %, 72,8–89,3). En 2016, les pays dans lesquels la prévalence de l'insuffisance d'activité physique atteignait ou dépassait 90 % étaient au nombre de 27 pour les filles, contre 2 pour les garçons.

Interprétation La majorité des adolescents ont un niveau d'activité physique qui ne répond pas aux critères fixés dans les lignes directrices actuelles. Des politiques et des programmes ayant fait leurs preuves doivent de toute urgence être appliqués à plus grande échelle pour améliorer le niveau d'activité des adolescents. L'investissement et le leadership sont indispensables à tous les niveaux pour remédier aux multiples facteurs et inégalités susceptibles de perpétuer la faible participation aux activités physiques et la disparité constatée entre les sexes, mobiliser les jeunes, et renforcer ainsi les occasions de pratiquer une activité physique dans toutes les communautés. De telles mesures permettront d'améliorer la santé de la jeune génération actuelle, ainsi que des générations futures, et contribueront à la réalisation des objectifs de développement durable à l'horizon 2030.
