# Supplementary material for: Global trends in insufficient physical activity among adolescents: a pooled analysis of 298 population-based surveys with 1·6 million participants
Source: Lancet Child Adolesc Health. 2020 Jan;4(1):23–35. doi: 10.1016/S2352-4642(19)30323-2 (PMC6919336; doi:10.1016/S2352-4642(19)30323-2)
Supplement: Spanish translation of the abstract [file mmc2.pdf]

THE LANCET
Child & Adolescent Health

Supplementary appendix 2

This translation in Spanish was submitted by the authors and we reproduce it as supplied. It has not been peer reviewed. *The Lancet*'s editorial processes have only been applied to the original in English, which should serve as reference for this manuscript.

Supplement to: Guthold R, Stevens GA, Riley LM, Bull FC. Global trends in insufficient physical activity among adolescents: a pooled analysis of 298 population-based surveys with 1·6 million participants. *Lancet Child Adolesc Health* 2019; published online Nov 21. https://doi.org/10.1016/S2352-4642(19)30323-2.

Los autores nos proporcionaron esta traducción al español y la reproducimos tal como nos fue entregada. No la hemos revisado. Los procesos editoriales de *The Lancet* se han aplicado únicamente al original en inglés, que debe servir de referencia para este manuscrito.

Tendencias mundiales en cuanto a actividad física insuficiente entre adolescentes: análisis conjunto de 298 encuestas poblacionales con 1·6 millones de participantes.

Tendencias mundiales en cuanto a actividad física insuficiente entre adolescentes: análisis conjunto de 298 encuestas poblacionales con 1,6 millones de participantes

Resumen

Antecedentes La actividad física conlleva muchos beneficios para la salud de los jóvenes. En 2018, la OMS puso en marcha un nuevo plan de acción sobre actividad física (Más personas activas para un mundo más sano) que incluía nuevas metas de reducción relativa de la prevalencia de actividad física insuficiente en un 15% para 2030 entre los adolescentes y los adultos. En el artículo describimos la prevalencia y tendencias actuales en cuanto a actividad física insuficiente entre adolescentes de 11 a 17 años en edad escolar por país y región, y en el ámbito mundial.

Métodos Realizamos un análisis conjunto de los datos de encuestas transversales que se compilaron en muestreos aleatorios con muestras integradas por al menos 100 individuos, representativas de una población nacional o subnacional definida, y que revelaron la prevalencia de actividad física insuficiente, desglosada por sexo, en adolescentes. La prevalencia tuvo que notificarse para al menos 3 de los años del grupo etario de 10 a 19 años. Calculamos la prevalencia de actividad física insuficiente en los adolescentes de 11 a 17 años en edad escolar (combinados y desglosados por sexo) en los diferentes países, los cuatro grupos de ingresos del Banco Mundial, nueve regiones y en todo el mundo para los años 2001-2016. Para derivar una definición normalizada de actividad física insuficiente y hacer ajustes en la cobertura de las encuestas realizadas solo en zonas urbanas, utilizamos modelos de regresión. Calculamos las tendencias en el tiempo utilizando modelos multinivel de efectos mixtos.

Resultados Utilizamos datos procedentes de 298 encuestas escolares realizadas en 146 países, territorios y zonas, con participación de 1,6 millones de alumnos de 11 a 17 años. En el ámbito mundial, en 2016, el 81,0% (intervalo de incertidumbre del 95% 77,8-87,7) de los alumnos entre 11 y 17 años de edad no realizaban actividad física suficiente (el 77,6% [76,1-80,4] de los niños y el 84,7% [83,0-88,2] de las niñas). Pese a que la prevalencia descendió significativamente entre 2001 y 2016 en el caso de los niños (80,1% [78,3-81,6] en 2001), en las niñas no se registraron cambios significativos (85,1% [83,1-88,0] en 2001). No había una pauta clara según el grupo de países por ingresos: la prevalencia de actividad insuficiente en 2016 era del 84,9% (82,6-88,2) en los países de ingresos bajos, el 79,3% (77,2-87,5) en los países de ingresos medianos bajos, el 83,9% (79,5-89,2) en los países de ingresos medianos altos y el 79,4% (74,0-86,2) en los países de ingresos altos. La región con mayor prevalencia de actividad insuficiente en 2016 era la de Asia y el Pacífico (países de ingresos altos) tanto para los niños (89,0%, 62,8-92,2) como para las niñas (95,6%, 73,7-97,9). Las regiones con menor prevalencia eran los países occidentales de ingresos altos para los niños (72,1%, 71,1-73,6), y Asia meridional para las niñas (77,5%, 72,8-89,3). En 2016, 27 países tenían una prevalencia de actividad insuficiente del 90% o superior para las niñas, mientras que para los niños esta situación se daba en dos países.

Interpretación La mayoría de los adolescentes no cumplen lo indicado en las directrices en vigor sobre actividad física. Es necesario reforzar urgentemente la aplicación de políticas y programas de eficacia conocida a fin de incrementar la actividad entre los adolescentes. La inversión y el liderazgo a todos los niveles para intervenir en las múltiples causas e inequidades que pueden perpetuar la baja participación en actividades físicas y las diferencias por sexo, así como la colaboración de la juventud misma, serán cruciales para fortalecer las oportunidades de actividad física en todas las comunidades. La adopción de dichas medidas permitirá mejorar la salud de las generaciones actuales y futuras y apoyar el logro de los Objetivos de Desarrollo Sostenible de 2030.
