# Supplementary material for: Global trends in insufficient physical activity among adolescents: a pooled analysis of 298 population-based surveys with 1·6 million participants
Source: Lancet Child Adolesc Health. 2020 Jan;4(1):23–35. doi: 10.1016/S2352-4642(19)30323-2 (PMC6919336; doi:10.1016/S2352-4642(19)30323-2)
Supplement: Supplementary appendix 3 [file mmc3.pdf]

THE LANCET
Child & Adolescent Health

Supplementary appendix 3

This appendix formed part of the original submission and has been peer reviewed. We post it as supplied by the authors.

Supplement to: Guthold R, Stevens GA, Riley LM, Bull FC. Global trends in insufficient physical activity among adolescents: a pooled analysis of 298 population-based surveys with 1·6 million participants. *Lancet Child Adolesc Health* 2019; published online Nov 21. https://doi.org/10.1016/S2352-4642(19)30323-2.

List of countries, territories and areas included in the analysis, by region

**Region (number of countries, territories and areas included in analysis)**	**Countries, territories and areas**
Central and Eastern Europe (16)	Albania; Bulgaria; Croatia; Czech Republic; Estonia; Hungary; Latvia; Lithuania; Moldova; North Macedonia; Poland; Romania; Russia; Slovakia; Slovenia; Ukraine
Central Asia, Middle East and North Africa (19)	Algeria; Armenia; Bahrain; Egypt; Iraq; Jordan; Kuwait; Lebanon; Libya; Mongolia; Morocco; Occupied Palestinian territory, including east Jerusalem; Oman; Qatar; Syria; Tunisia; Turkey; United Arab Emirates; Yemen
East and South East Asia (14)	Brunei; Cambodia; China; Taiwan; Indonesia; Laos; Malaysia; Maldives; Myanmar; Philippines; Sri Lanka; Thailand; Timor-Leste; Viet Nam
High-income Asia Pacific (2)	Singapore; South Korea
High-income Western countries (25)	Australia; Austria; Belgium; Canada; Denmark; Greenland; Finland; France; Germany; Greece; Iceland; Ireland; Israel; Italy; Luxembourg; Malta; Netherlands; New Zealand; Norway; Portugal; Spain; Sweden; Switzerland; UK; USA
Latin America and Caribbean (32)	Anguilla; Antigua and Barbuda; Argentina; Bahamas; Barbados; Belize; Bolivia; Brazil; British Virgin Islands; Cayman Islands; Chile; Colombia; Costa Rica; Dominica; Ecuador; El Salvador; Grenada; Guatemala; Guyana; Honduras; Mexico; Montserrat; Netherlands Antilles; Paraguay; Peru; Saint Kitts and Nevis; Saint Lucia; Saint Vincent and the Grenadines; Suriname; Trinidad and Tobago; Uruguay; Venezuela
Oceania (16)	American Samoa; Cook Islands; Fiji; French Polynesia; Guam; Kiribati; Nauru; Niue; Palau; Samoa; Solomon Islands; Tokelau; Tonga; Tuvalu; Vanuatu; Wallis and Futuna
South Asia (6)	Afghanistan; Bangladesh; Bhutan; India; Nepal; Pakistan
Sub-Saharan Africa (16)	Benin; Botswana; Djibouti; Ghana; Kenya; Mauritania; Mauritius; Mozambique; Namibia; Senegal; Seychelles; Sudan; Tanzania; Uganda; Zambia; Zimbabwe

Data sources used in the analysis, by country, territory and area

Country, territory and area	Year	Sample size	Survey/Study	Age range	Urban only coverage	Coverage (where non national)
Afghanistan	2014	2155	GSHS	11-17		
Albania	2014	4924	HBSC	11-15		
Algeria	2011	4405	GSHS	11-16		
American Samoa	2011	2841	YRBS	14-17		
Anguilla	2016	767	GSHS	12-17		
Antigua and Barbuda	2009	1178	GSHS	12-16		
Argentina	2007	1877	GSHS	12-16		
Argentina	2012	27034	GSHS	11-16		
Armenia	2010	2833	HBSC	11-15		
Armenia	2014	3680	HBSC	11-15		
Australia	2009	12064	NaSSDA	14-17		
Australia	2011	1686	NHS	12-17		
Australia	2012	2800	NNPAS	11-17		
Austria	2001	4366	HBSC	11-15		
Austria	2005	4775	HBSC	11-15		
Austria	2010	5003	HBSC	11-15		
Austria	2014	3420	HBSC	11-15		
Bahamas	2013	1298	GSHS	12-16		
Bahrain	2016	7017	GSHS	11-17		
Bangladesh	2014	2881	GSHS	12-17		
Barbados	2011	1550	GSHS	12-16		
Belgium	2001	10612	HBSC	11-15		Flemish part
Belgium	2005	8787	HBSC	11-15		
Belgium	2010	8192	HBSC	11-15		
Belgium	2014	10240	HBSC	11-15		
Belize	2011	2001	GSHS	11-16		
Benin	2009	2650	GSHS	11-16		
Benin	2015	1562	GSHS	12-17		
Bhutan	2016	6043	GSHS	11-17		
Bolivia	2012	3425	GSHS	11-16		
Botswana	2005	2087	GSHS	12-16		
Brazil	2012	109104	PeNSE	13-16		
Brazil	2015	16600	PeNSE	13-17		
British Virgin Islands	2009	1627	GSHS	11-16		
Brunei	2014	2490	GSHS	12-17		
Bulgaria	2005	4854	HBSC	11-15		
Bulgaria	2014	4796	HBSC	11-15		
Cambodia	2013	2892	GSHS	12-17		
Canada	2001	4361	HBSC	11-15		
Canada	2005	5787	HBSC	11-15		

Country, territory and area	Year	Sample size	Survey/Study	Age range	Urban only coverage	Coverage (where non national)
Canada	2010	15710	HBSC	11-15		
Cayman Islands	2007	1218	GSHS	12-16		
Chile	2004	8002	GSHS	12-16		Metropolitan Region, Region I, Region V, Region VIII
Chile	2005	3577	GSHS	12-16		Metropolitan Region, Region VIII
Chile	2013	1871	GSHS	12-17		
China	2003	8791	GSHS	11-16	urban	Beijing, Hangzhou, Wuhan, Wurumqi
Taiwan	2012	6753	GSHS	12-16		
Colombia	2005	7490	ENSIN	13-17		
Colombia	2007	9524	GSHS	12-16	urban	Bogota, Valledupar, Manizales, Cali, Bucaramanga
Cook Islands	2011	1247	GSHS	12-16		
Cook Islands	2015	642	GSHS	13-17		
Costa Rica	2009	2637	GSHS	12-16		
Croatia	2001	4366	HBSC	11-15		
Croatia	2005	4965	HBSC	11-15		
Croatia	2010	6252	HBSC	11-15		
Croatia	2014	5740	HBSC	11-15		
Czechia	2001	5012	HBSC	11-15		
Czechia	2005	4775	HBSC	11-15		
Czechia	2010	4404	HBSC	11-15		
Czechia	2014	5056	HBSC	11-15		
Denmark	2001	4587	HBSC	11-15		
Denmark	2005	5682	HBSC	11-15		
Denmark	2010	4046	HBSC	11-15		
Denmark	2014	3846	HBSC	11-15		
Greenland	2001	873	HBSC	11-15		
Greenland	2005	1358	HBSC	11-15		
Greenland	2010	1205	HBSC	11-15		
Djibouti	2007	1706	GSHS	11-16		
Dominica	2009	1523	GSHS	11-16		
Ecuador	2007	5264	GSHS	11-16	urban	Guayaquil, Quito, Zamora
Egypt	2006	5112	GSHS	11-16		
Egypt	2011	2445	GSHS	11-16		
El Salvador	2013	1845	GSHS	12-16		
Estonia	2001	3976	HBSC	11-15		

Country, territory and area	Year	Sample size	Survey/Study	Age range	Urban only coverage	Coverage (where non national)
Estonia	2005	4477	HBSC	11-15		
Estonia	2010	4224	HBSC	11-15		
Estonia	2014	4052	HBSC	11-15		
Fiji	2010	2784	GSHS	11-16		
Fiji	2015	2916	GSHS	13-17		
Finland	2001	5348	HBSC	11-15		
Finland	2005	5193	HBSC	11-15		
Finland	2010	6607	HBSC	11-15		
Finland	2014	5838	HBSC	11-15		
France	2001	8185	HBSC	11-15		
France	2005	7141	HBSC	11-15		
France	2010	6128	HBSC	11-15		
France	2014	5636	HBSC	11-15		
French Polynesia	2016	2787	GSHS	12-17		
Germany	2001	5635	HBSC	11-15		
Germany	2005	7224	HBSC	11-15		
Germany	2010	4955	HBSC	11-15		
Germany	2014	5910	HBSC	11-15		
Ghana	2007	5949	GSHS	11-16		
Ghana	2012	2378	GSHS	11-17		
Greece	2001	3807	HBSC	11-15		
Greece	2005	3690	HBSC	11-15		
Greece	2010	4899	HBSC	11-15		
Greece	2014	4114	HBSC	11-15		
Grenada	2008	1431	GSHS	12-16		
Guam	2013	1303	YRBS	14-17		
Guatemala	2009	5323	GSHS	11-16		
Guatemala	2015	4070	GSHS	12-17		
Guyana	2004	1170	GSHS	12-16		
Guyana	2010	2278	GSHS	13-16		
Honduras	2012	1695	GSHS	11-16		
Hungary	2001	4057	HBSC	11-15		
Hungary	2005	3498	HBSC	11-15		
Hungary	2010	4787	HBSC	11-15		
Hungary	2014	3876	HBSC	11-15		
Iceland	2005	9476	HBSC	11-15		
Iceland	2010	11049	HBSC	11-15		
Iceland	2014	10440	HBSC	11-15		
India	2007	7744	GSHS	11-16		
Indonesia	2007	3074	GSHS	12-16		
Indonesia	2015	10706	GSHS	11-17		
Iraq	2012	1962	GSHS	11-16		

Country, territory and area	Year	Sample size	Survey/Study	Age range	Urban only coverage	Coverage (where non national)
Ireland	2001	2875	HBSC	11-15		
Ireland	2005	4840	HBSC	11-15		
Ireland	2010	4724	HBSC	11-15		
Ireland	2014	4078	HBSC	11-15		
Israel	2001	5341	HBSC	11-15		
Israel	2005	5350	HBSC	11-15		
Israel	2014	6194	HBSC	11-15		
Italy	2001	4357	HBSC	11-15		
Italy	2005	3920	HBSC	11-15		
Italy	2010	4811	HBSC	11-15		
Italy	2014	4010	HBSC	11-15		
Jordan	2004	2361	GSHS	13-16		
Jordan	2007	2133	GSHS	12-16		
Kenya	2003	3428	GSHS	11-16		
Kiribati	2011	1551	GSHS	12-16		
Kuwait	2011	2633	GSHS	12-16		
Kuwait	2015	3053	GSHS	12-17		
Laos	2015	3585	GSHS	13-17		
Latvia	2001	3455	HBSC	11-15		
Latvia	2005	4221	HBSC	11-15		
Latvia	2010	4264	HBSC	11-15		
Latvia	2014	5536	HBSC	11-15		
Lebanon	2011	2189	GSHS	11-16		
Lebanon	2016	4954	GSHS	11-17		
Libya	2007	2128	GSHS	11-16		
Lithuania	2001	5644	HBSC	11-15		
Lithuania	2005	5632	HBSC	11-15		
Lithuania	2010	5323	HBSC	11-15		
Lithuania	2014	5732	HBSC	11-15		
Luxembourg	2005	4300	HBSC	11-15		
Luxembourg	2010	4072	HBSC	11-15		
Luxembourg	2014	3112	HBSC	11-15		
Malaysia	2012	25081	GSHS	11-17		
Maldives	2009	2980	GSHS	11-16		
Malta	2001	1945	HBSC	11-15		
Malta	2005	1389	HBSC	11-15		
Malta	2014	2258	HBSC	11-15		
Mauritania	2010	1951	GSHS	11-16		
Mauritius	2007	2246	GSHS	12-16		
Mauritius	2011	2147	GSHS	12-16		
Mexico	2016	1472	ENSANUT	11-14		
Mongolia	2010	5137	GSHS	11-16		

Country, territory and area	Year	Sample size	Survey/Study	Age range	Urban only coverage	Coverage (where non national)
Moldova	2014	4650	HBSC	11-15		
Mongolia	2013	5121	GSHS	11-17		
Montserrat	2008	197	GSHS	13-16		
Morocco	2006	2499	GSHS	12-16		
Morocco	2010	2784	GSHS	11-16		
Morocco	2016	5727	GSHS	11-17		
Mozambique	2015	1325	GSHS	11-17		
Myanmar	2007	2773	GSHS	12-16		
Myanmar	2016	2752	GSHS	11-17		
Namibia	2004	5925	GSHS	11-16		
Namibia	2014	3304	GSHS	11-17		
Nauru	2011	485	GSHS	12-16		
Nepal	2015	6167	GSHS	11-17		
Netherlands	2001	4268	HBSC	11-15		
Netherlands	2005	4228	HBSC	11-15		
Netherlands	2010	4520	HBSC	11-15		
Netherlands	2014	4236	HBSC	11-15		
Netherlands Antilles	2015	2087	GSHS	11-17		
New Zealand	2007	9098	The Youth07 survey	13-17		
New Zealand	2012	8202	The Youth12 survey	13-17		
Niue	2010	106	GSHS	12-16		
North Macedonia	2001	4030	HBSC	11-15		
North Macedonia	2005	5271	HBSC	11-15		
North Macedonia	2007	1978	GSHS	12-17		
North Macedonia	2010	3897	HBSC	11-15		
North Macedonia	2014	4162	HBSC	11-15		
Norway	2001	5015	HBSC	11-15		
Norway	2005	4697	HBSC	11-15		
Norway	2010	4338	HBSC	11-15		
Norway	2014	3050	HBSC	11-15		
Occupied Palestinian territory, including east Jerusalem	2010	4302	GSHS	11-16		
Oman	2005	2862	GSHS	12-16		
Oman	2010	1500	GSHS	13-16		
Oman	2015	3090	GSHS	11-17		
Pakistan	2009	5112	GSHS	11-16		
Palau	2011	304	YRBS	15-17		
Paraguay	2016	3150	GSHS	11-17		
Peru	2010	2827	GSHS	12-16		
Philippines	2003	6840	GSHS	11-16		
Philippines	2007	5539	GSHS	12-16		
Philippines	2011	5074	GSHS	12-16		

Country, territory and area	Year	Sample size	Survey/Study	Age range	Urban only coverage	Coverage (where non national)
Philippines	2015	8263	GSHS	11-17		
Poland	2001	6310	HBSC	11-15		
Poland	2005	5489	HBSC	11-15		
Poland	2010	4241	HBSC	11-15		
Poland	2014	4518	HBSC	11-15		
Portugal	2001	2928	HBSC	11-15		
Portugal	2005	3919	HBSC	11-15		
Portugal	2010	4036	HBSC	11-15		
Portugal	2014	4990	HBSC	11-15		
Qatar	2011	1829	GSHS	11-16		
Romania	2005	4684	HBSC	11-15		
Romania	2010	5352	HBSC	11-15		
Romania	2014	3942	HBSC	11-15		
Russia	2001	8032	HBSC	11-15		
Russia	2005	8231	HBSC	11-15		
Russia	2010	5174	HBSC	11-15		
Russia	2014	4576	HBSC	11-15		
Saint Kitts and Nevis	2011	1674	GSHS	13-16		
Saint Lucia	2007	1246	GSHS	12-16		
Saint Vincent and the Grenadines	2007	1225	GSHS	12-16		
Samoa	2011	2114	GSHS	11-16		
Senegal	2005	3059	GSHS	11-16		
Seychelles	2007	1350	GSHS	11-17		
Seychelles	2015	2439	GSHS	11-17		
Singapore	2015	9151	Students' Health Survey	13-17		
Slovakia	2005	3877	HBSC	11-15		
Slovakia	2010	5281	HBSC	11-15		
Slovakia	2014	6016	HBSC	11-15		
Slovenia	2001	3915	HBSC	11-15		
Slovenia	2005	5119	HBSC	11-15		
Slovenia	2010	5429	HBSC	11-15		
Slovenia	2014	4984	HBSC	11-15		
Solomon Islands	2011	1296	GSHS	11-16		
South Korea	2012	62063	KYRBS	13-17		
South Korea	2013	60423	KYRBS	13-17		
Spain	2001	5823	HBSC	11-15		
Spain	2005	8891	HBSC	11-15		
Spain	2010	5040	HBSC	11-15		
Spain	2014	11138	HBSC	11-15		
Sri Lanka	2008	2552	GSHS	12-16		
Sri Lanka	2016	3192	GSHS	12-17		

Country, territory and area	Year	Sample size	Survey/Study	Age range	Urban only coverage	Coverage (where non national)
Sudan	2012	2063	GSHS	12-16		
Suriname	2009	1632	GSHS	12-16		
Suriname	2016	1867	GSHS	12-17		
Sweden	2001	3896	HBSC	11-15		
Sweden	2005	4392	HBSC	11-15		
Sweden	2010	6645	HBSC	11-15		
Sweden	2014	7656	HBSC	11-15		
Switzerland	2001	4528	HBSC	11-15		
Switzerland	2005	4579	HBSC	11-15		
Switzerland	2010	6611	HBSC	11-15		
Switzerland	2014	6530	HBSC	11-15		
Syria	2010	3045	GSHS	11-16		
Tanzania	2006	2094	GSHS	11-16	urban	Dar-Es-Salaam
Tanzania	2014	3561	GSHS	11-17		
Thailand	2008	2726	GSHS	12-16		
Thailand	2012	1160	Physical Activity Surveillance System	11-17		
Thailand	2013	1160	Physical Activity Surveillance System	11-17		
Thailand	2014	1160	Physical Activity Surveillance System	11-17		
Thailand	2015	1160	Physical Activity Surveillance System	11-17		
Thailand	2015	5575	GSHS	11-17		
Thailand	2016	1160	Physical Activity Surveillance System	11-17		
Timor-Leste	2015	2965	GSHS	11-17		
Tokelau	2014	66	GSHS	11-15		
Tonga	2010	2157	GSHS	12-16		
Trinidad and Tobago	2007	1144	GSHS	12-16		
Trinidad and Tobago	2011	956	GSHS	11-16		
Tunisia	2008	2783	GSHS	11-16		
Turkey	2005	5552	HBSC	11-15		
Turkey	2010	5574	HBSC	11-15		
Tuvalu	2012	870	GSHS	12-16		
Uganda	2003	2986	GSHS	11-16		
UK	2001	14314	HBSC	11-15		England, Scotland, Wales
UK	2005	15309	HBSC	11-15		England, Scotland, Wales
UK	2010	15652	HBSC	11-15		England, Scotland, Wales
UK	2014	16244	HBSC	11-15		England, Scotland, Wales
Ukraine	2001	4090	HBSC	11-15		
Ukraine	2005	5069	HBSC	11-15		

Country, territory and area	Year	Sample size	Survey/Study	Age range	Urban only coverage	Coverage (where non national)
Ukraine	2010	5890	HBSC	11-15		
Ukraine	2014	4552	HBSC	11-15		
United Arab Emirates	2005	15189	GSHS	11-16		
United Arab Emirates	2010	2484	GSHS	12-16		
United Arab Emirates	2016	5329	GSHS	11-17		
USA	2001	5025	HBSC	11-15		
USA	2005	3892	HBSC	11-15		
USA	2010	6274	HBSC	11-15		
USA	2011	14915	YRBSS	14-17		
USA	2013	10910	YRBSS	14-17		
USA	2015	15046	YRBSS	14-17		
Uruguay	2006	3336	GSHS	12-16		
Uruguay	2012	3420	GSHS	13-16		
Vanuatu	2011	1084	GSHS	11-16		
Venezuela	2003	4276	GSHS	11-16	urban	Barinas, Lara
Viet Nam	2013	3038	GSHS	13-17		
Wallis and Futuna	2015	991	GSHS	11-17		
Yemen	2008	1058	GSHS	11-16		
Yemen	2014	2219	GSHS	11-17		
Zambia	2004	1871	GSHS	11-16		
Zimbabwe	2003	5368	GSHS	11-16		Bulawayo, Harare, Manicaland

List of countries, territories and areas with trend data

Argentina; Armenia; Australia; Austria; Belgium; Benin; Brazil; Bulgaria; Canada; Chile; Colombia; Cook Islands; Croatia; Czech Republic; Denmark; Greenland; Egypt; Estonia; Fiji; Finland; France; Germany; Ghana; Greece; Guatemala; Guyana; Hungary; Iceland; Indonesia; Ireland; Israel; Italy; Jordan; Kuwait; Latvia; Lebanon; Lithuania; Luxembourg; Malta; Mauritius; Mongolia; Morocco; Myanmar; Namibia; Netherlands; New Zealand; North Macedonia; Norway; Oman; Philippines; Poland; Portugal; Romania; Russia; Seychelles; Slovakia; Slovenia; South Korea; Spain; Sri Lanka; Suriname; Sweden; Switzerland; Tanzania; Thailand; Trinidad and Tobago; Turkey; Ukraine; United Arab Emirates; UK; USA; Uruguay; Yemen.

Detailed methods description

Objective

To produce comparable country estimates for adolescent boys and girls, and both sexes combined, for the prevalence of insufficient physical activity, defined as not meeting current WHO recommendations on physical activity for health, which is, doing less than 60 minutes of moderate to vigorous physical activity per day (1).

We documented these estimates as recommended by the Guidelines for Accurate and Transparent Health Estimates Reporting (GATHER) (2).

Inclusion criteria

The criteria for data to be included in the comparable country estimates on insufficient physical activity for adolescents were as follows:

- The definition of insufficient physical activity had to be:
    - Less than 60 minutes of moderate to vigorous-intensity physical activity per day, OR
    - Less than 60 minutes of moderate to vigorous-intensity physical activity per day on less than 5 days per week;
- Data had to be collected from the general adolescent population or the school going population through random sampling, and be representative of a national or defined subnational population;
- Data had to be based on self-report, rather than objectively measured physical activity;
- Data had to capture adolescent ages of 10-19 years, or at least 3 years of this age group (such as 13-15 year olds);
- Prevalence values had to be presented along with sample size, as well as clear description of survey methodology;
- Survey sample size had to be at least n=100.

Note: since all surveys that met the inclusion criteria were conducted in the school setting, the estimates were produced and labelled for "school going adolescents".

Data search

Data were searched through:

- Existing WHO databases at Headquarters and Regional Offices;
- Communication with WHO Regional focal points, researchers and experts;
- Verification of the results of the 2017 NCD Country Capacity Survey (CCS) (3) *[Survey question: Have surveys of risk factors (may be a single risk factor or multiple) been conducted in your country for any of the following: […] Physical Inactivity. Was there a survey on adolescents? […] When was the last survey conducted? […]];*
- A non-systematic literature search in PubMed up to December, 2016, focused on countries with no data, using the search terms *("Physical activity" OR "exercise" OR "inactivity") AND ("adolescents" OR "adolescence" OR "young people" OR "youth") AND ("Global School-based Student Health Survey" OR "GSHS" OR "Health Behaviour in School-aged Children" OR "HBSC" OR "Youth Risk Behavior Survey" OR "YRBS" OR "school survey" OR "school-based survey") [AND "individual country name"];*
- Communication through personal networks;
- An on-line country consultation from 31 July – 8 Sept 2017.

Data sources

Two main data sources met the inclusion criteria, as well as additional national surveys: the Global School-based Student Health Survey (GSHS), the Health Behaviour among School-aged Children (HBSC) that's conducted in many European countries, the USA and Canada, the Youth Risk Behaviour Surveys (YRBS) undertaken in the USA and its territories American Samoa and Guam, as well as in Palau, South Korea's KYRBS, the Australian NaSSDA, the Brazilian PeNSE, the Colombian ENSIN, the Mexican ENSANUT, the Thai Physical Activity Surveillance System, and Singapore's Students' Health Survey. Overall, 298 surveys were included in the estimation process, representing data from 146 countries (table 1).

To assess physical activity behaviour, most of the included sources, including the GSHS and the HBSC, used a question that was originally developed by Prochaska et al (4) and further adapted, reading *"During the past 7 days, on how many days were you physically active for a total of at least 60 minutes per day?"*, with answer options ranging from 0 to 7 days. Students were typically asked to add up all the time they spent in any kind of physical activity each day, and were provided examples on what activities to include.

Table 1. Distribution of data sources across World Bank income groups (5).

	All countries	World Bank income group	Low income	Lower middle income	Upper middle income	High income
Total	231	215	31	51	58	75
With data	146	141	8	37	40	56
% with data	63.2	65.6	25.8	72.5	69.0	74.7

Definition adjustment

For surveys where only the definition of "Less than 60 minutes of moderate to vigorous-intensity physical activity per day on less than 5 days per week" was available, an adjustment was made in order to derive the target definition of "Less than 60 minutes of moderate to vigorous-intensity physical activity per day". This was done using results from surveys providing both definitions, with the following regression model:

Y (Prevalence of "Less than 60 minutes of moderate to vigorous-intensity physical activity per day") = $\alpha + \beta_1$*(Prevalence of "Less than 60 minutes of moderate to vigorous-intensity physical activity per day on less than 5 days per week") + β_2*(sex_code) + β_3*(age) + β_4*(edu_years) + ε
where sex_code was a code for male and female, age was the age in years of each of the observations, edu_years was the mean years of education, and ε was the error term assumed normally distributed.

```
. regress fail_meet_recs less_than_5 sex age edu_years

      Source |       SS           df       MS      Number of obs   =     1,714
-------------+----------------------------------   F(4, 1709)      =   1089.30
       Model |  76945.4271         4  19236.3568   Prob > F        =    0.0000
    Residual |  30179.7858     1,709  17.6593246   R-squared       =    0.7183
-------------+----------------------------------   Adj R-squared   =    0.7176
       Total |  107125.213     1,713    62.53661   Root MSE        =    4.2023

------------------------------------------------------------------------------
fail_meet_~s |      Coef.   Std. Err.      t    P>|t|     [95% Conf. Interval]
-------------+----------------------------------------------------------------
 less_than_5 |   .5272296   .0095215    55.37   0.000     .5085545    .5459046
         sex |    2.45605   .2177535    11.28   0.000     2.028959    2.883142
         age |   .1451809   .0591219     2.46   0.014     .0292219    .2611398
   edu_years |   .5123158   .0410493    12.48   0.000     .4318037    .5928279
       _cons |   35.12726   1.142859    30.74   0.000     32.88571    37.36881
------------------------------------------------------------------------------
```

Adjustment for coverage

141 out of the 146 countries reporting sex- and age-specific prevalence of insufficient physical activity had nationally representative data. Only subnational data including urban and rural areas were available from two countries, and urban data only were available for China, Ecuador, and Venezuela (Bolivarian Republic of). In addition to national data, Colombia and the United Republic of Tanzania also had urban-only surveys. To derive a national estimate for surveys that covered urban areas only, the relationship between insufficient physical activity in urban versus rural areas was explored using information from surveys where estimates for both urban and rural areas existed, and by applying the following regression model:

Y (Prevalence of "Less than 60 minutes of moderate to vigorous-intensity physical activity per day" (rural)) = $\alpha + \beta_1$*("Less than 60 minutes of moderate to vigorous-intensity physical activity per day" (urban)) + β_2*(sex_code) + β_3*(age) + β_4*(edu_years) + ε

where sex_code was a code for male and female, age was the age in years of each of the observations, edu_years was the mean years of education, and ε was the error term assumed normally distributed.

```
. regress fail_meet_recs_rural fail_meet_recs_urban sex age edu_years

      Source |       SS           df       MS      Number of obs   =        57
-------------+----------------------------------   F(4, 52)        =     12.05
       Model |  973.590288         4  243.397572   Prob > F        =    0.0000
    Residual |   1050.3122        52  20.1983115   R-squared       =    0.4810
-------------+----------------------------------   Adj R-squared   =    0.4411
       Total |  2023.90249        56  36.1411158   Root MSE        =    4.4943

--------------------------------------------------------------------------------------
fail_meet_recs_rural |      Coef.   Std. Err.      t    P>|t|     [95% Conf. Interval]
---------------------+----------------------------------------------------------------
fail_meet_recs_urban |   .2339066   .0743201     3.15   0.003     .0847725    .3830408
                 sex |   3.630713   1.326825     2.74   0.008     .9682441    6.293182
                 age |  -.0388537   .3523706    -0.11   0.913    -.7459371    .6682296
           edu_years |  -.8111758    .300703    -2.70   0.009    -1.414581   -.2077711
               _cons |   66.77163   7.519964     8.88   0.000     51.68172    81.86155
--------------------------------------------------------------------------------------
```

After deriving an estimate for rural areas for surveys with urban data only, the urban and rural estimates were combined using estimated population data for the year of estimation of the United Nations Population Division (6).

Age range
The available data sources included the minimum age of 11, and the maximum age of 17 years. The two main data sources GSHS and HBSC covered the following age groups: the HBSC 11, 13 and 15 year olds, and the GSHS focused on 13-15 year olds until 2012, and then expanded the focus age range to 13-17 years. However, since GSHS sampling includes entire classes, some students that were outside the focus age range were also included in each survey. Appendix 2 provides the age range for each survey included.

For each country and survey, the available single year age groups were aggregated into one final estimate, representing 11-17 year olds, by using country population figures as estimated by the United Nations Population Division (Revision 2016) (6) for the respective survey year.

Time trends
Data included in the estimation process were from the years 2001 – 2016, with 73 of the 146 countries having two or more surveys from different years. Using these trend data, time trends were estimated for all countries with data.

To estimate prevalence values for missing years, the following multilevel mixed-effects linear regression model was applied for both sexes separately:

Y (Logit of the prevalence of not meeting "WHO PA recs") $_{ij} = \beta_0 + \beta_1 (\text{year})_{ij} + \beta_2(\text{super-region})_{ij} + \beta_3(\text{perc\_urban})_{ij} + u_{0j} + u_{1j} (\text{year})_{ij} + \varepsilon_{ij}$
Where j = countries, i = years, super-region was a variable combining countries into nine regions, as previously used in other, similar analysis (6), perc_urban was the percentage of urban population in each country at the respective years, uj was the random effect occurring at the country level, allowing for a random slope on year, and ε ij was the error term.

```
. mixed logit_fail_meet_recs year i.super-region perc_urban ||
whoname:year, covariance(unstructured)

Mixed-effects ML regression                     Number of obs     =        297
Group variable: whoname                         Number of groups  =        146

                                                Obs per group:
                                                              min =          1
                                                              avg =        2.0
                                                              max =          6

                                                Wald chi2(10)     =      46.93
Log likelihood = -91.787237                     Prob > chi2       =     0.0000

------------------------------------------------------------------------------------------------
          logit_fail_meet_recs |      Coef.   Std. Err.      z    P>|z|     [95% Conf. Interval]
-------------------------------+----------------------------------------------------------------
                          year |  -.0039424   .0038264    -1.03   0.303     -.011442    .0035572
                               |
                BMI_superregion |
   Central and Eastern Europe  |  -.2817129   .1118425    -2.52   0.012    -.5009201   -.0625056
     East and South East Asia  |   .2503451   .1345874     1.86   0.063    -.0134414    .5141315
     High-income Asia Pacific  |   .3299972    .291739     1.13   0.258    -.2418007    .9017952
High-income Western countries  |  -.1241418   .1025785    -1.21   0.226    -.3251919    .0769083
  Latin America and Caribbean  |   .1014152   .1051597     0.96   0.335     -.104694    .3075245
                      Oceania  |   .2019561   .1324277     1.53   0.127    -.0575974    .4615095
                   South Asia  |   .0388946   .1935987     0.20   0.841    -.3405519    .4183412
           Sub-Saharan Africa  |   .3120924   .1279402     2.44   0.015     .0613342    .5628507
                               |
                    perc_urban |  -.0007001   .0015555    -0.45   0.653    -.0037488    .0023486
                         _cons |   9.310902   7.678912     1.21   0.225    -5.739489    24.36129
------------------------------------------------------------------------------------------------

------------------------------------------------------------------------------
  Random-effects Parameters  |   Estimate   Std. Err.     [95% Conf. Interval]
-----------------------------+------------------------------------------------
whoname: Unstructured        |
                   var(year) |   .0001501   .0002325      7.20e-06    .0031253
                  var(_cons) |   593.4568   936.4356      26.93081    13077.63
             cov(year,_cons) |  -.2984048   .4665575     -1.212841    .6160312
-----------------------------+------------------------------------------------
               var(Residual) |   .0550837   .0082325      .0410967     .073831
------------------------------------------------------------------------------
LR test vs. linear model: chi2(3) = 96.80                 Prob > chi2 = 0.0000
```

Final country estimates

For countries with data, estimates were produced for all years 2001 – 2016. For countries with no survey, no estimate was produced.

Estimates for World Bank Income groups and regions

To produce estimates for World Bank income groups, we used groupings for the year 2016 (5), and for regional estimates, we used previously defined country groupings used in similar analyses (7, 8). For all different groupings, country estimates were weighted by population size of each country, using country population figures as estimated by the United Nations Population Division (Revision 2016) (6). Countries with no estimate were not taken into account.

Uncertainty

Uncertainty around the country estimates was computed by using the predicted values and standard errors (combining standard errors of fixed and random effects) of the hierarchical trend models.

For the estimates for World Bank Income groups, regions, and the world, confidence intervals were produced using the bootstrap methodology: for men and women separately, we ran the hierarchical trend model 1000 times, using a random 80% of all survey data each time. We then produced World Bank Income group, regional, and global prevalence estimates for each of the 1000 datasets. Of these 1000 results for each subgroup, gender and year, we took the $2{\cdot}5^{th}$ and the $97{\cdot}5^{th}$ percentile to form the confidence interval around the estimate. To test for significance of trends and sex

differences in those subgroups and globally, based on a previously described approach (8, 9), we calculated the difference in prevalence between 2001 and 2016 and between sexes for each draw and considered a change in prevalence over time or between sexes to be statistically significant if at least 97.5% of draws showed a changing trend or a sex difference in the same direction.

Consultation with WHO Member States

A consultation with WHO Member States was carried out from 31 July to 8 September 2017. Data submitted by Member States during that period that met inclusion criteria were included in a re-run of the model during October 2017.

References

(1) World Health Organization. Global recommendations on physical activity for health. Geneva, Switzerland: World Health Organization 2010. Available at: http://whqlibdoc.who.int/publications/2010/9789241599979_eng.pdf Accessed on 1 Nov 2018.

(2) Stevens GA, Alkema L, Black RE, et al. Guidelines for Accurate and Transparent Health Estimates Reporting: the GATHER statement. Lancet 2016;388:e19-e23.

(3) World Health Organization. Assessing national capacity for the prevention and control of NCDs. http://www.who.int/ncds/surveillance/ncd-capacity/en/ (accessed 10 July 2019).

(4) Prochaska JJ, Sallis JF, Long B. A physical activity screening measure for use with adolescents in primary care. Arch Pediatr Adolesc Med 2001;155:554-9.

(5) The World Bank. List of economies, December 2016. Available at: databank.worldbank.org/data/download/site-content/CLASS.xls Accessed 13 May 2018.

(6) United Nations. Department of Economic and Social Affairs. Population Division. Monitoring Global Population Trends. Available at: http://www.un.org/en/development/desa/population/publications/index.shtml Accessed 13 October 2018.

(7) NCD Risk Factor Collaboration (NCD-RisC). Worldwide trends in body-mass index, underweight, overweight, and obesity from 1975-2016: a pooled analysis of 2416 population-based measurement studies in 128.9 million children, adolescents, and adults. Lancet 2017;S0104-6736(17)32129-3.

(8) Guthold R, Stevens GA, Riley LM, Bull FC. Worldwide trends in insufficient physical activity from 2001 to 2016: a pooled analysis of 358 population-based surveys with 1.9 million participants. The Lancet Global Health 2018; **6**(10): e1077-e86.

(9) Stevens GA, White RA, Flaxman SR, et al. Global prevalence of vision impairment and blindness: magnitude and temporal trends, 1990-2010. Ophthalmology 2013;120(12):2377-2384.

Figure 5

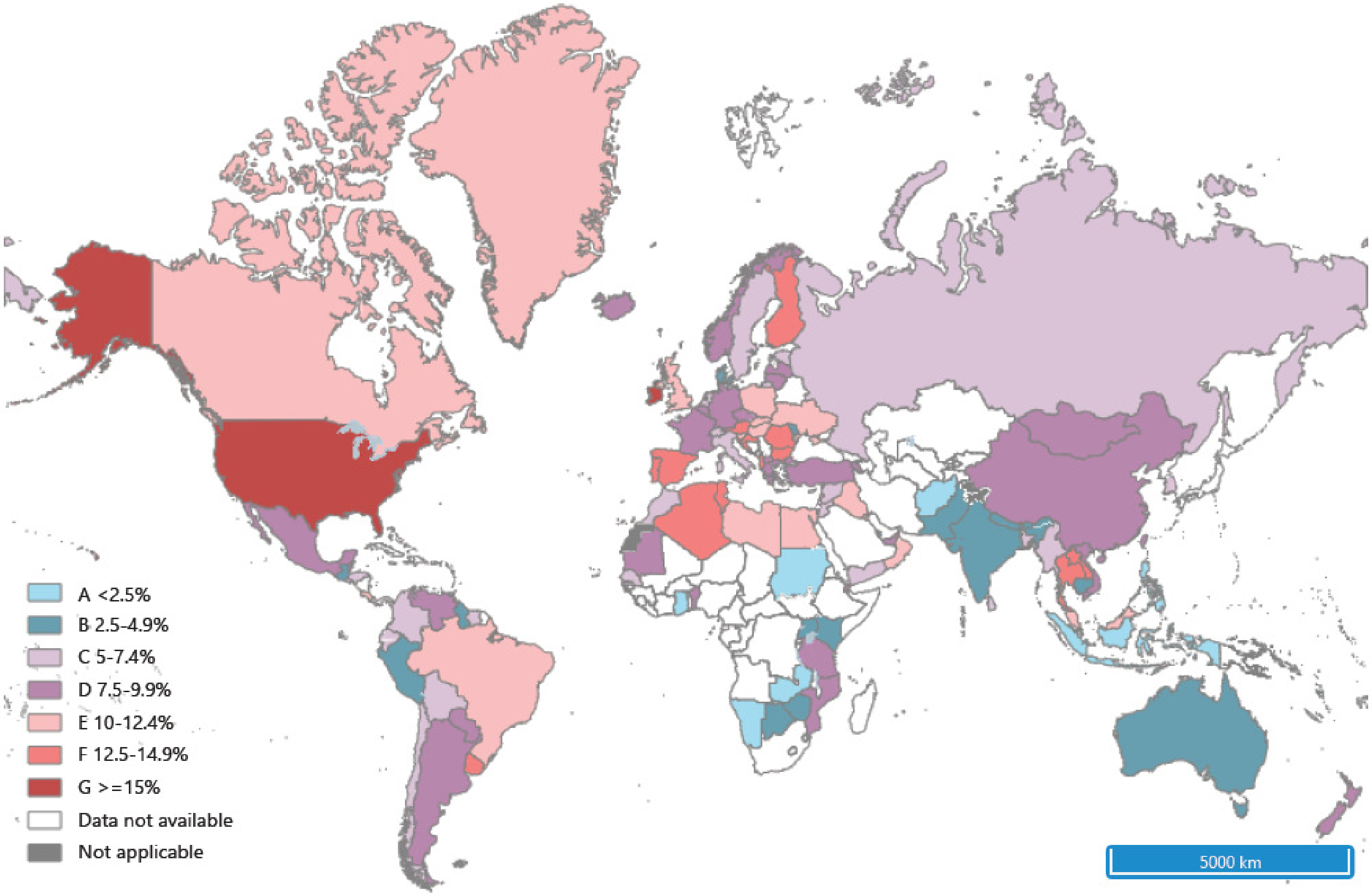

Figure 5. Sex difference in prevalence of insufficient physical activity among school going adolescents, 11-17 years, 2016. Female prevalence greater than male prevalence in all countries except Afghanistan, Samoa, Tonga and Zambia.
